# Disparities in Acceptance of Deceased Donor Kidneys Between the United States and France and Estimated Effects of Increased US Acceptance

**DOI:** 10.1001/jamainternmed.2019.2322

**Published:** 2019-08-26

**Authors:** Olivier Aubert, Peter P. Reese, Benoit Audry, Yassine Bouatou, Marc Raynaud, Denis Viglietti, Christophe Legendre, Denis Glotz, Jean-Phillipe Empana, Xavier Jouven, Carmen Lefaucheur, Christian Jacquelinet, Alexandre Loupy

**Affiliations:** 1 Paris Translational Research Center for Organ Transplantation, INSERM, UMR-S970, Paris, France; 2 Department of Kidney Transplantation, Necker Hospital, Assistance Publique - Hôpitaux de Paris, Paris, France; 3 Department of Medicine, Renal-Electrolyte and Hypertension Division, University of Pennsylvania, Philadelphia; 4 Center for Clinical Epidemiology and Biostatistics, Perelman School of Medicine, University of Pennsylvania, Philadelphia; 5 Agence de la Biomédecine, Saint Denis la Plaine, France; 6 Department of Nephrology and Kidney Transplantation, Saint-Louis Hospital, Assistance Publique - Hôpitaux de Paris, Paris, France; 7 INSERM U1018, CESP, Université Paris Sud, Villejuif, France

## Abstract

**Importance:**

Approximately 3500 donated kidneys are discarded in the United States each year, drawing concern from Medicare and advocacy groups.

**Objective:**

To estimate the effects of more aggressive allograft acceptance practices on the donor pool and allograft survival for the population of US wait-listed kidney transplant candidates.

**Design, Setting, and Participants:**

A nationwide study using validated registries from the United States and France comprising comprehensive cohorts of deceased donors with organs offered to kidney transplant centers between January 1, 2004, and December 31, 2014. Data were analyzed between September 1, 2018, and April 5, 2019.

**Main Outcomes and Measures:**

The primary outcome was kidney allograft discard. The secondary outcome was allograft failure after transplantation. We used logistic regression to model organ acceptance and discard practices in both countries. We then quantified using computer simulation models the number of kidneys discarded in the United States that a more aggressive system would have instead used for transplantation. Finally, based on actual survival data, we quantified the additional years of allograft life that a redesigned US system would have saved.

**Findings:**

In the United States, 156 089 kidneys were recovered from deceased donors between 2004 and 2014, of which 128 102 were transplanted, and 27 987 (17.9%) were discarded. In France, among the 29 984 kidneys recovered between 2004 and 2014, 27 252 were transplanted, and 2732 (9.1%, *P* < .001 vs United States) were discarded. The mean (SD) age of kidneys transplanted in the United States was 36.51 (17.02) years vs 50.91 (17.34) years in France (*P* < .001). Kidney quality showed little change in the United States over time (mean [SD] kidney donor risk index [KDRI], 1.30 [0.48] in 2004 vs 1.32 [0.46] in 2014), whereas a steadily rising KDRI in France reflected a temporal trend of more aggressive organ use (mean [SD] KDRI, 1.37 [0.47] in 2004 vs 1.74 [0.72] in 2014; *P* < .001). We applied the French-based allocation model to the population of US deceased donor kidneys and found that 17 435 (62%) of kidneys discarded in the United States would have instead been transplanted under the French system. We further determined that a redesigned system with more aggressive organ acceptance practices would generate an additional 132 445 allograft life-years in the United States over the 10-year observation period.

**Conclusions and Relevance:**

Greater acceptance of kidneys from deceased donors who are older and have more comorbidities could provide major survival benefits to the population of US wait-listed patients.

**Trial Registration:**

ClinicalTrials.gov identifier: NCT03723668

## Key Points

**Question:**

Would a more aggressive approach to organ acceptance provide a benefit to wait-listed kidney transplant candidates?

**Findings:**

This cohort study analyzes the use of 156 089 deceased donor kidneys in the United States and 29 984 in France and finds that the US discard rate of these kidneys is nearly twice that of France. It uses computer simulation to model a lower US discard rate similar to that of France, and estimates a US increase of 132 445 allograft life-years.

**Meaning:**

Greater acceptance of kidneys from older and comorbid deceased donors in the United States could provide major survival benefits to the population of wait-listed patients.

## Teaser

This cohort study evaluates the number of kidney transplants that would have taken place in the United States if the US acceptance rate of deceased donor kidneys was as high as that of France and uses computer simulation algorithms to measure the potential gains in allograft survival years if US programs adopted less restrictive kidney acceptance practices.

## Introduction

The lack of organs for kidney transplantation is a major public health problem owing to its attributable mortality and excess cost to health care systems while wait-listed patients are maintained on chronic dialysis.^1^ Nearly 5000 people in the United States and more than 3000 people in Europe die each year while waiting for a kidney transplant.^2,3^ Yet, in the United States, over 3500 donated kidneys are discarded annually.^4^

Over the past decade, the worldwide scarcity of donated kidneys and the decline in the number of living donor transplants in some countries have prompted a variety of efforts to expand the organ supply, such as accepting organs from donors who were older or had comorbidities or other injuries.^5,6,7,8^ As new treatment approaches have developed to manage the complications of kidney transplantation, recent studies have suggested that compared with dialysis, even the lowest-quality kidneys lengthen the life span, on average, for transplant recipients.^9,10,11^

The leading explanations for the high rate of kidney discard include the intense regulatory scrutiny of US transplant programs, which may lose credentials if their 1-year death and graft failure outcomes exceed predicted outcomes.^5^ Schold et al^12^ reported that transplant programs that received negative reports performed 22.4 fewer transplants subsequently vs a mean increase of 7.8 transplants for other programs (*P* = .001). Financial disincentives may also discourage acceptance of lower-quality kidneys because managing complications such as delayed graft function prolongs hospitalisation.^13,14^ An additional concern is the role of kidney biopsies as a flawed method of assessing allograft quality. Although biopsies can yield information about scarring, acute kidney injury, or chronic disease, biopsies may also promote needless discard if the pathologic analysis is completed by individuals who lack sufficient time and skill.^15,16^

Two major initiatives from the United Network for Organ Sharing (UNOS), the organization responsible for organ allocation in the United States, failed to improve the kidney acceptance rate. First, UNOS introduced the Kidney Donor Risk Index (KDRI) for all kidney offers in 2012. The KDRI is a score that predicts survival of deceased donor kidneys based on 10 donor characteristics and was intended to simplify the process of judging organ quality for clinicians.^17^ A lower KDRI score indicates better kidney quality. Second, in 2014, UNOS changed the kidney allocation system so that lower-quality kidneys are offered over wider geographic areas. Despite these initiatives, the number of discarded kidneys rose from 2127 (14.9%) in 2006 to 3631 (20%) in 2016.^18^

In this context, the experience of transplant programs outside the United States could offer novel approaches to making organ utilization more efficient through the examination of the disposition of organs that are usually discarded in the United States. Indeed, long waiting times and aging populations are characteristic of both European and US transplant programs. To facilitate the use of older organs, Eurotransplant implemented the Senior Program, matching kidneys from donors older than 65 years to recipients in the same age group.^19,20^ France, Spain, and Italy, which are outside the Eurotransplant region, have followed similar policies to progressively increase the donor pool by offering and using a wider range of deceased donors for transplantation. The French transplant system in particular offers a useful contrast because French transplant programs face less regulatory scrutiny than US programs and do not use donor kidney biopsies in organ acceptance decisions.

Evidence is scarce regarding the hypothetical benefit of transplanting kidneys that would have been discarded otherwise in the United States. Therefore, we evaluated the number of kidney transplants that would have taken place using data from a nationwide cohort study in the United States and France, and used computer simulation algorithms to measure the potential gains in allograft survival years that would result if US programs adopted less restrictive kidney acceptance practices.

## Methods

### Study Population

The trial protocol is available in Supplement 1. We assembled a cohort of all consecutive kidneys recovered for the purpose of transplantation from donors deceased from brain death or circulatory death between January 1, 2004, and December 31, 2014. We also examined outcomes for the recipients of kidney transplants from these donors in the United States and in France. In both countries, we excluded living donor kidney transplants, multiorgan transplant recipients, kidneys that were offered to transplant centers but were never recovered, and patients with missing data to calculate the KDRI score (eFigure 1A and B in Supplement 2).

### Data Source

#### Organ Procurement and Transplantation Network in the United States

Data on the donors and kidney transplant recipients were obtained using registry data from the Organ Procurement and Transplantation Network (OPTN). The OPTN data system includes data on all donors, wait-listed candidates, and transplant recipients in the United States, as submitted by the members of the Organ Procurement and Transplantation Network. The Health Resources and Services Administration (HRSA) of the US Department of Health and Human Services oversees the activities of the OPTN contractor. The kidney allocation policies are described in detail elsewhere.^4^

#### CRISTAL Registry in France

Data on the donors and recipients in the French cohort were obtained from the national CRISTAL registry, initiated in 1996 and maintained by the *Agence de la Biomédecine*, which prospectively collects data on all potential donors and organ transplant candidates, along with their outcomes.^21,22^ By law, data collection is provided by all organ procurement organizations and transplant centers in France. The University of Pennsylvania determined that the proposal met eligibility criteria for institutional review board review exemption because all data are anonymized and studies using the data do not require institutional review board approval. The study was conducted according to French law stating that research studies based on the CRISTAL national registry are part of transplant assessment activity and do not require institutional review board approval. Kidney allocation policies are described in detail in https://www.agence-biomedecine.fr/.^23^

### Kidney Donor Risk Index Assessment

Organ quality was estimated by calculating the KDRI and the Kidney Donor Profile Index (KDPI), with lower values suggestive of better quality. The KDRI and KDPI are currently used as part of the OPTN allocation system for deceased donor kidneys in the United States and have been validated as reliable measures of organ quality in the United States and other developed countries.^17,24,25^ We calculated the KDRI as described by the OPTN (eMethods 1.2 in Supplement 2).

### Outcomes

The primary outcome was donor kidney discard, both single and dual (the latter refers to instances in which both kidneys were procured, and neither was transplanted). The secondary outcome was allograft failure after transplantation (defined as the need for renal replacement therapy or preemptive retransplantation). Death-censored and non–death-censored graft survival were considered in the analyses (eMethods in Supplement 2).

### Statistical Analysis

Continuous variables were described using the means and standard deviations (SDs) or medians and interquartile ranges (IQRs). We compared the means and proportions between groups using the student *t* test, analysis of variance (ANOVA), or the χ^2^ test, as appropriate.

#### Statistical Modeling of Kidney Allograft Discard Practice in Each Country

We used US and French data to estimate changes in the number of kidneys transplanted and discarded in the United States where French organ acceptance practices applied to organs donated in the United States, and vice versa.

The association between the (log-transformed) KDRI score and kidney allograft discard was assessed through logistic regression analyses. A score was generated using the regression coefficient of the model, and a probability of discard using this score was generated for each patient. Model calibration using the KDRI was assessed by graphical methods and discrimination by the receiver operating characteristic (ROC) curve and area under the curve (AUC; eMethods 1.1.1 in Supplement 2).

The Monte-Carlo simulation method was used to estimate the number of kidneys that would have been discarded using the KDRI-driven kidney acceptance practice between countries. Acceptance practices were simulated for each kidney separately. For each kidney (*k*) of a given deceased donor (*d*) from 1 of our 2 countries, the following procedures were performed: (1) a uniform 0-to-1 random number *U_dk_* was generated; and (2) its probability *P_dk_* of being discarded was computed from the other-country logistic regression model according to the actual KDRI *K_d_* of the donor, and the kidney *k* was virtually discarded if and only if U*_dk _*≤ *P_dk_* (eMethods 1.1.3 in Supplement 2).^26^ We considered that the size of the deceased donor cohorts (156 089 for the United States and 29 984 for France) offered such a wide range of variability that no bootstrapping techniques were needed.^27^

#### Quantification of Predicted Kidney Allograft Survival of Discarded Kidneys

Stratified by country of transplantation, we performed kidney survival analysis from the time of transplantation until a maximum follow-up of 10 years, with kidney graft loss as the event of interest, defined as the patient’s return to dialysis or retransplantation. Ten groups were generated using the KDRI deciles, and allograft survival was generated for each decile. For the patients who died with a functioning graft, graft survival was censored at death.^28^ Kidney allograft survival was plotted using Kaplan-Meier curves and compared using the log-rank test. The mean allograft survival for each KDRI decile was estimated using the restricted mean survival time (RMST) (the area under the Kaplan-Meier curves) in the survRM2 package in R (version 3.2.1, R Foundation for Statistical Computing) among transplanted patients.

#### Estimation of the Gain or Loss in Allograft Life-Years Based on Observed Survival Data

Using actual survival data, we then quantified the change in the number of years of allograft life that would have been saved or lost under a redesigned allocation system (for example, if kidney acceptance practices were modified) based on the allograft acceptance patterns in the other country. This number corresponds to the survival time of the kidneys that would have been saved or lost by mimicking the kidney acceptance practices of the other country and using the mean survival time associated with those kidneys.

We used STATA statistical software (version 14, STATA Corp) and R (version 3.2.1, R Foundation) for the descriptive and survival analyses. All statistical tests were 2-sided, and *P* values <.05 were considered significant.

## Results

### Characteristics of Kidneys Recovered for Transplantation in France and in the United States

The French cohort included 15 500 deceased donors between January 1, 2004 and December 31, 2014, from whom 29 984 kidneys were recovered. Among these recovered kidneys, 27 252 were transplanted and 2732 (9.1%) were discarded.

The US cohort included 78 517 deceased donors between 2004 and 2014, from whom 156 089 kidneys were recovered for transplantation. Among these recovered kidneys, 128 102 were transplanted and 27 987 (17.9%) were discarded (eFigure 1A and B in Supplement 2).

Table 1 presents the characteristics of transplanted and discarded kidneys in France compared with those in the United States. Among transplanted kidneys, the mean (SD) donor age was 50.91 (17.34) years in France vs 36.51 (17.02) years in the United States (*P* < .001). In the United States, fewer donors had hypertension (n = 31 720 [24.76%] vs 7919 [29.06%] in France; *P* < .001) or died of cerebrovascular causes (n = 41 858 [32.68%] vs 14 871 [54.57%] in France; *P* < .001). Kidneys in France were less likely to be donated after cardiac death (prevalence rate of 1.6% vs 11.7% in the United States) and to come from donors seropositive for hepatitis C virus (prevalence rate of 0.1% vs 2.1% in the United States). In France, the discard rate was lower for weekend procurement (667 kidneys discarded during the weekend [8.5%] vs 2065 discarded during weekdays [9.3%, *P* = .02]). In the United States, the overall discard rate was similar during the weekdays and weekend, with 20 237 kidneys discarded during weekdays (prevalence rate of 17.9%) vs 7750 discarded during the weekend (prevalence rate of 18.0%, *P* = .70). In the United States, there was a higher discard rate of kidneys recovered from African American donors (4748 of 22 751; 20.9%) compared with non–African American donors (23 239 of 133 338; 17.43%, *P *< .001).

**Table 1.** Baseline Characteristics of the Transplanted and Discarded Kidneys in the US and French Systems^a^

Characteristic	Transplanted Kidneys		*P* Value^b^	Discarded Kidneys		*P* Value^b^
	US Cohort (n=128 102), No. (%)	French Cohort (n=27 252), No. (%)		US Cohort (n=27 987), No. (%)	French Cohort (n=2732), No. (%)	
Age, mean (SD), y	36.51 (17.02)	50.91 (17.34)	<.001	52.15 (16.39)	61.58 (17.04)	<.001
Male sex	77 695 (60.65)	16 110 (59.11)	<.001	14 694 (52.50)	1710 (62.59)	<.001
Height, mean (SD), cm	167.76 (20.68)	169.61 (11.25)	<.001	167.28 (19.23)	167.47 (15.16)	.79
Weight, mean (SD), kg	77.18 (24.81)	73.40 (16.16)	<.001	81.32 (25.41)	75.16 (19.20)	<.001
BMI, mean (SD)	26.68 (6.64)	25.39 (4.99)	<.001	28.44 (7.32)	26.48 (5.49)	<.001
Hypertension	31 720 (24.76)	7919 (29.06)	<.001	16 801 (60.03)	1349 (49.38)	<.001
Diabetes mellitus	8085 (6.31)	1844 (6.77)	.005	6203 (22.16)	379 (13.87)	<.001
Hypertension and diabetes	5495 (4.29)	1204 (4.42)	.35	5243 (18.74)	310 (11.35)	<.001
Cerebrovascular death	41 858 (32.68)	14 871 (54.57)	<.001	16 106 (57.55)	1816 (66.47)	<.001
DCD donor	15 037 (11.74)	438 (1.61)	<.001	3816 (13.63)	199 (7.28)	<.001
Creatinine, mean (SD), mg/dL	1.11 (0.88)	1.02 (0.61)	<.001	1.50 (1.14)	1.23 (0.80)	<.001
KDRI, mean (SD)	1.23 (0.41)	1.50 (0.58)	<.001	1.83 (0.56)	2.03 (0.72)	<.001
KDPI, mean (SD)	0.45 (0.28)	0.60 (0.29)	<.001	0.77 (0.23)	0.80 (0.22)	<.001
Positive HCV serology	2712 (2.12)	36 (0.13)	<.001	2932 (10.48)	29 (1.06)	<.001
African American donors	18 003 (14.05)	NA^c^	NA	4748 (16.97)	NA^c^	NA

Abbreviations: BMI, body mass index (calculated as weight in kilograms divided by height in meters squared); DCD, donors with cardiac death; KDPI, Kidney Donor Risk Profile; KDRI, Kidney Donor Risk Index; NA, not applicable.

^a^ Data are based on national registry data from the Organ Procurement and Transplantation Network for the United States and from the national CRISTAL registry in France maintained by the *Agence de la Biomédecine*, which prospectively collect information on all potential donors and organ transplant recipients along with their outcomes. Inclusion criteria included kidneys from donors with available KDRI data elements. The number of participants with missing data for KDRI was 1525 (1%) for the United States and 1403 (4.5%) for France.

^b^ χ^2^ tests were conducted for the comparison of proportions, and *t* tests were conducted for the comparison of continuous variables.

^c^ Reporting of ethnicity for donors is not permitted per the French regulation data protection rules.

**Figure 1.**
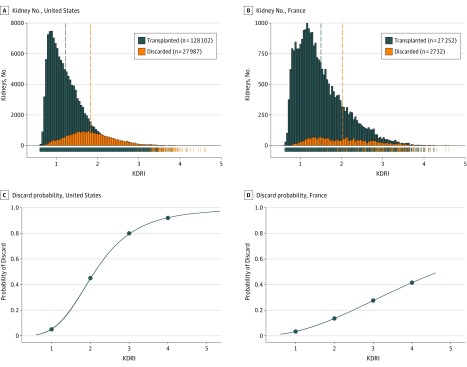
Deceased Donor Kidneys Transplanted and Discarded in the United States and France Between 2004 and 2014 and Their Kidney Donor Risk Index (KDRI) Scores^a^ Data are based on 156 089 recovered kidneys in the United States, including 128 102 transplanted and 27 987 discarded kidneys, and on 29 984 recovered kidneys in France, including 27 252 transplanted and 2732 discarded kidneys. A, The distribution of KDRI scores for transplanted (blue) and discarded (orange) kidneys in the United States. B, The distribution of the KDRI score for transplanted (blue) and discarded (orange) kidneys in France. Dashed vertical lines correspond to the mean KDRI of transplanted kidneys (dashed blue) and discarded (dashed orange) kidneys. C, The probability of discard in the United States by KDRI; and D, The probability of discard in France by KDRI. The blue curve corresponds to the probability of discard according to the KDRI in the United States (C) and in France (D). ^a^Lower KDRI indicates better kidney quality.

The mean (SD) KDRI of transplanted deceased donors was significantly higher in France (1.50 [0.58]) than in the United States (1.23 [0.41]) (*P* < .001) (Figure 1A and B). Similar trends were observed using the KDPI score (Table 1) (eFigure 2 in Supplement 2).

### Temporal Trends in Organ Quality and Age for Transplanted Deceased Donor Kidney Allografts

From 2004 through 2014, there was a steadily rising KDRI in France (mean [SD] 1.37 [0.47] in 2004 and 1.74 [0.72] in 2014, *P* < .001), reflecting a temporal trend of more aggressive organ use, whereas the quality of kidneys transplanted in the United States showed little change (mean [SD] KDRI, 1.30 [0.48] in 2004 and 1.32 [0.46] in 2014) (eFigure 3A and B in Supplement 2). Similar trends were observed using the KDPI score (eFigure 4 in Supplement 2). This higher KDRI in France was driven principally by increasing donor age (mean [SD] age, 56.17 [18.32] years in France vs 39.08 [17.27] years in the United States in 2014) (eFigure 3C and D in Supplement 2).

### Prediction Models for Kidney Discard Decisions in France and the United States

The KDRI (log transformation) was highly associated with the discard of the kidney in France and in the United States (OR, 3.88; 95% CI, 3.83-3.93; in the United States; OR, 2.18; 95% CI, 2.07-2.30; *P *< .001 in France). The 2 models showed good accuracy, with an AUC of 0.82 for the United States model, and an AUC of 0.72 for the French model (eFigure 5A in Supplement 2); both models displayed excellent calibration (eFigure 5B and C in Supplement 2). These analyses revealed that the US allocation system had a higher probability of discarding kidneys with higher KDRIs than in France (Figure 1C and D). As revealed in Figure 1, a KDRI of 1, 2, 3, and 4 results in actual kidney discard rates of 5%, 45%, 80%, and 92%, respectively, in the United States, compared with 3%, 13%, 27%, and 42% discard rates in France, respectively.

**Figure 2.**
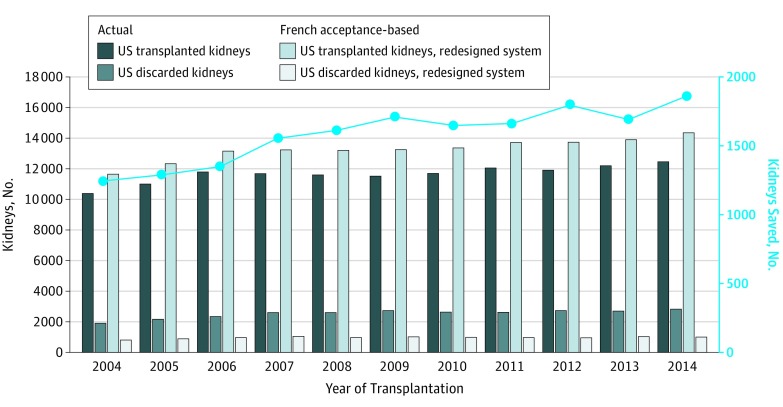
Number of Actually Transplanted and Discarded Kidneys in the United States Contrasted With the Number of Kidneys That Would Have Been Saved and Discarded Using a Redesigned System The number of actually transplanted and discarded kidneys in the United States between 2004 and 2014 and the kidneys that would have been transplanted and discarded if the French model had been applied in the United States. The blue line shows the number of kidneys that would have been saved in the United States if the French model had been applied. Overall, a total of 17 435 US kidneys would have been saved during the observation period.

We then applied the French allocation model to the US cohort and demonstrated that a French-based practice pattern translated to an estimated 10 552 discarded kidneys compared with the actual number of 27 987 discarded kidneys in the United States (French model-based discard rate of 6.8% compared with actual discard rate of 17.9% in the United States, *P* < .001). Overall, the application of the French-based discard practice pattern would have corresponded to an estimated 17 435 fewer discarded kidneys (62.3% among all discarded kidneys) in the United States during the observation period from 2004 to 2014 (Figure 2).

### Estimation of the Potential Gain in Allograft Life-Years If Kidney Discard Rates Were Reduced in the United States

Overall, the observed allograft survival for deceased donor kidneys transplanted in the United States was 95.0%, 83.5%, 77.3%, and 67.3% at 1, 5, 7, and 10 years posttransplant, respectively. Based on outcomes for actual US kidney recipients, we determined the mean allograft survival by 10-year posttransplantation and stratified allograft survival according to the 10 deciles of the KDRI score (eFigure 6 and eFigure 7 in Supplement 2; stratified allograft survival according to the 10 deciles of KDRI score for French kidney recipients is shown in eFigure 8 in Supplement 2).

**Figure 3.**
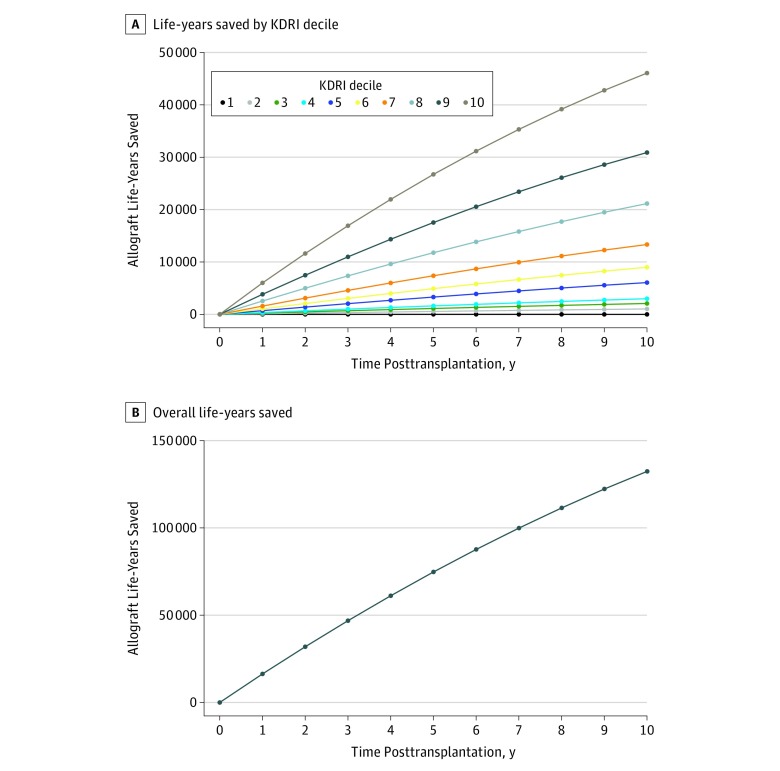
Estimation of the Allograft Life-Years Gained From Reducing Kidney Discard Rates in the United States Through a Redesigned System KDRI Indicates Kidney Donor Risk Index. The greatest gain in life-years is achieved through reduced discard of the lowest-quality kidneys. A, The life-years saved by decile of the Kidney Donor Risk Index (KDRI) by applying French acceptance-based patterns to the pool of US kidneys. B, The life-years saved overall if organ acceptance patterns in the United States had followed the French acceptance model.

We then assessed the kidney survival of the pool of kidneys discarded in the United States that would have been accepted at French transplant centers. Based on the US allograft survival data, we calculated that this pool of discarded kidneys in the United States would have provided 132 445 potential allograft life-years by 10 years posttransplantation (Figure 3).

### Sensitivity Analysis

#### Estimation of the Number of Donated Kidneys That Would Be Discarded Using Donor Variables Instead of the Single KDRI Score

Instead of using the single KDRI score, we performed bivariate and multivariable analyses of the association between individual donor variables and the outcome of kidney discard for the United States (eTable 1A and B in Supplement 2) and for France (eTable 2A and B in Supplement 2). The AUC of the final model was 0.82 (95% CI, 0.82-0.82) and 0.74 (95% CI, 0.73-0.75) for the US- and French-based models, respectively, showing similar results to the original model that included only the single KDRI score as a covariate. This model revealed similar projections of discard rates as those found in primary analyses.

#### Estimation of Non–Death-Censored Allograft Life-Years If Kidney Discard Rates Were Reduced in the United States

We subsequently used non–death-censored allograft survival analysis (instead of death-censored analyses) to estimate the number of allograft life-years saved if more aggressive organ transplantation practices were applied to kidneys donated in the United States. This analysis offers the benefit of taking into account the effect of recipient deaths on allograft outcomes and was based on observed survival times for actual recipients of similar kidneys in the United States. The kidney allograft survival times according to the KDRI deciles are shown in eFigure 9 in Supplement 2. (Non–death-censored allograft survival according to the KDRI deciles in France are shown in eFigure 10 in Supplement 2). When we applied the kidney discard prediction models, the potential gain in allograft life-years in the United States would have been 109 079 years during the 10-year observation period (eFigure 11 in Supplement 2).

#### Estimation of the Reduction in Donor Pool and Loss in Allograft Life-Years If the US-Based Kidney Acceptance Model Were Applied to France (Vice Versa Analysis)

We next evaluated the lost allograft life-years by applying the model of US-based acceptance practices to the French cohort. Applying the US model to the French allocation system resulted in an increase of 7558 discarded kidneys (discard rate of 25.2%) compared with the actual number of 2732 discarded kidneys in France (actual discard rate of 9.1%, *P* < .001). The graft survival of this pool of kidneys that would have been discarded if US acceptance practices were applied to the French system was: 87.5%, 83.4%, and 76.3% at 5, 7, and 10 years, respectively.

Based on the French allograft survival data, we calculated that the pool of hypothetically discarded kidneys from the US-based acceptance model, but actually transplanted kidneys in France would have experienced a loss of 41 977 allograft life years over 10 years if kidney acceptance practice patterns from the United States had been adopted.

### Subgroup Analyses

Various subgroup analyses were performed to test the robustness and generalizability and to account as best as possible for differences between the French and American donor populations. As shown in Table 2, the results of these subgroup analyses were similar to primary analyses in terms of the magnitude of associations as well as the discard and allograft survival.

**Table 2.** Subgroup Analyses Involving Year of Organ Recovery, Donor Ethnicity, Donor Hepatitis C Virus Serostatus, and Donation After Cardiac Death Status

Variable	Transplanted Kidneys, No.	Discarded Kidneys, No.	Actual Discard Rate, %	Discard Rate in the Redesigned System, %	Potential Gain of Allograft Life-Years in the Redesigned System, y
Overall US data set (2004-2014)	128 102	27 987	17.9	6.8	132 445
Year of Organ Recovery					
US donors, y					
2004-2008	56 360	11 737	17.2	6.9	53 282
2009-2014	71 742	16 250	18.5	6.7	79 163
Characteristics of US donors					
Non-African American	110 099	23 239	17.4	6.3	113 264
Hepatitis C negative	125 266	25 018	16.6	6.6	113 825
Non-DCD donors	113 065	24 171	17.6	6.8	112 381
Non–African American, hepatitis C negative, and non-DCD donors	93 721	17 668	15.8	6.2	81 267

Abbreviation: DCD, donors with cardiac death. These analyses show the discard rate and the potential gain in allograft life-years in a redesigned organ acceptance system among donor subgroups in the United States to account for the differences between the French and American donor populations.

## Discussion

The high rate of discard of kidneys recovered for transplantation in the United States suggests the potential to gain insight into allocation efficiency from other health care systems. In this comparison of kidneys recovered from deceased donors from 2004 through 2014, the age of the donors of transplanted kidneys remained stable in the United States. By contrast, transplant practice in France addressed the unmet need for transplantable organs by accepting lower-quality kidneys from older donors, such that by 2014, the mean age of kidney donors was 56 years in France vs 39 years in the United States. Using real life survival data from 2 comprehensive registries, we calculated that elevated kidney discard rates in the United States led to the loss of 17 435 kidneys and 132 445 unrealized allograft life years during the observation period. This finding indicates that the US kidney transplantation system, which has driven innovations in donor utilization in the domains of kidneys obtained after cardiac death and from hepatitis C virus-infected donors, still has substantial potential for growth by accepting more organs from donors who are older and have comorbidities including diabetes and hypertension. Such growth would offer substantial benefit for wait-listed patients.^29^

This study builds on an important tradition of implementing international comparisons to identify best practices for treating patients with kidney disease. For 2 decades, the Dialysis Outcomes and Practice Patterns Study has generated robust studies of variation in dialysis treatment across 20 countries.^30^ Other investigators have documented wide international variation in the rate of organ donor registration^31^ and deceased organ donation,^32^ and thereby invigorated efforts to identify effective approaches from nations such as Spain, where donation rates are high. This study leverages data from the United States and France to show inefficiencies in the utilization of deceased donor kidneys in the United States and points the way to the solution. A systematic literature review (eMethods 1.3 in Supplement 2) revealed that no studies systematically assessed kidney discard rates between different countries and their impact in terms of gain or loss of opportunities for wait-listed patients by virtually applying an EU-based allocation approach to the US system. Given the paucity of evidence and the infeasibility of a randomized clinical trial, our study advances the field and brings appropriate analytical approaches to the problem.

Our results strongly suggest that patients wait-listed for kidney transplantation would benefit if US transplant programs were more willing to accept lower-quality kidneys, particularly from older deceased donors. Notably, the higher KDRI scores of kidneys transplanted in France vs the United States was primarily owing to older donor age and not to the wider use of kidneys from donors with other specific characteristics that may negatively impact allograft quality, such as hepatitis C seropositivity or cardiac death.^17^ If programs in France did use kidneys from donors with hepatitis C virus or cardiac death at the same rate as US programs do, average kidney allograft quality in France would be lower and the difference in kidney quality scores such as the KDRI between the 2 countries would grow even larger. Although older donor age is a risk factor for allograft failure, kidneys from donors in their 50s or 60s may nonetheless extend life for older transplant candidates in particular.^9,10,33^ Schold et al^34^ examined US registry data and determined that kidney transplant candidates older than 65 years lived longer if they reduced their waiting time by accepting kidneys from “extended criteria donors”—those older than 60 years or older than 50 years with comorbidities such as hypertension. Frei et al^19^ examined the Eurotransplant Senior Program (ESP) at German, Austrian, Dutch, and Belgian centers, which involves preferential allocation of elderly (>65 years) deceased donor kidneys into elderly recipients, and reported that ESP reduced waiting time to transplant by 5 months. Subsequent reports involving ESP recipients have suggested that posttransplant survival is approximately equivalent to remaining on dialysis.^20,35^ A study of 2040 kidney transplant candidates in Spain, among whom 389 received a kidney transplant from a deceased donor aged 75 years or older, also reported a survival benefit from using these older donor kidneys. At 1 year, the hazard of death across all age groups was approximately half the risk of remaining on dialysis (hazard ratio, 0.51; 95% CI, 0.34-0.77).^36^

Our models of the potential allograft life-years that could be gained from reducing organ discard in the United States may actually underestimate the lost opportunity. A tremendous need exists for viable kidneys suitable for older adults. The percentage of US kidney transplant recipients older than 60 years increased from 22% in 2004 to 32% in 2017, but more than 35 000 patients older than 60 years remained on the waiting list.^37^ Goldberg et al^38^ reported that, compared with eligible donor patients in the United States aged 18 to 39 years, eligible donors aged 55 to 64 years (odds ratio, 0.72; 95% CI, 0.67-0.77) and 65 years or younger (odds ratio, 0.58; 95% CI, 0.52-0.64) became organ donors at much lower rates. In summary, no organs are recovered from many potential older donors, perhaps because of the perception of a low probability than any transplant program would accept the organs. However, if transplant programs created the demand, organ procurement from older individuals might well rise. Because approximately 95 000 patients are currently waiting for kidney transplant in the United States, most transplant programs should not have difficulty finding willing older patients who would be appropriate candidates for higher-KDRI organs. To improve organ quality of marginal kidneys with higher KDRI, new therapeutic approaches are being developed. Those therapeutics include hemodynamic stabilization of brain-dead donors, optimized organ preservation techniques, refinement of organ allocation, and treatment of transplant recipients as recently described by Tullius et al.^8^ With the potential growth in the use of higher-KDRI organs in the future to respond to the organ shortage, better strategies of careful donor management are needed. These strategies include the use of pumping devices that are currently widely implemented in France.

### Limitations

This study has limitations. First, unmeasured confounding by donor characteristics not assessed in the KDRI, such as vascular disease, might contribute to international differences in organ discard rates. Second, the new organ allocation system has only been implemented in 2014, thus the analysis of data from 2004 to 2014 cannot assess if the new organ allocation system has impacted organ utilization. However, Stewart et al^6^ examined national registry data and showed that the kidney discard rate did not improve after 2014 when the KDRI and other modifications to kidney allocation were implemented. Third, although allografts of similar quality are more likely to be discarded in the United States than in France, posttransplant outcomes for lower-quality kidneys might also be worse in the United States.^39,40,41^ Nevertheless, our estimates of the gain in life-years if US centers became more aggressive with kidney acceptance were very substantial, whether based on survival data of similar kidneys transplanted in the United States or France. Fourth, US allocation policy does not preferentially allocate older kidneys to older recipients. As a result, greater procurement of high-KDRI kidneys might have adverse consequences if these kidneys were transplanted into young recipients.^20,42^ This highlights the need to select the best recipient for a given kidney from a deceased donor including an optimal pairing based on patient’s age and organ longevity.^8^ This problem could be overcome with refinements to allocation or education of transplant professionals. Finally, the overall kidney acceptance process, starting from a consented deceased donor to transplantation, may be subject to variations in practice patterns or data recording between countries.

Although this study does not offer a specific policy prescription to increase the acceptance of higher-KDRI kidneys in the United States, the findings offer valuable evidence about lost opportunities that transplant leaders can use to change policy and practice. For example, the results could motivate US regulatory organizations to incentivize wider use of higher-KDRI kidneys instead of focusing predominantly on 1-year posttransplant outcomes. Furthermore, this work of using higher-risk organs might be best pioneered by centers with sufficient expertise and ability to manage complications successfully.

## Conclusions

The high discard rate of deceased donor kidneys is a major concern for the US transplant field. We found that the age and KDRI of US deceased donor kidneys remained stable from 2004 to 2014 in the United States, whereas the French transplant system responded to the organ shortage by accepting lower-quality kidneys, especially those from older donors. Policies designed to enhance the acceptance of donated kidneys in the United States could drive meaningful increases in the number of kidney transplants and bring the benefits of transplantation to thousands of wait-listed patients.

## Notes

Supplement 1. **Trial Protocol**

Supplement 2. **eMethods** **eTable 1A.** US cohort: Donor characteristics associated with kidney discard bivariate analysis **eTable 1B.** US cohort: Donor characteristics associated with kidney discard multivariable analysis **eTable 2A.** French Cohort: Donor characteristics associated with kidney discard bivariate analysis **eTable 2B.** French Cohort: Donor characteristics associated with kidney discard in France in multivariable analysis **eFigure 1.** Flowchart of the study population in the US and in France **eFigure 2.** Distribution of deceased donor kidney quality as manifested in the Kidney Donor Risk Profile (KDPI) kidneys in the US and in France **eFigure 3.** Change in the mean deceased donor age and Kidney Donor Risk Index in the US and France between 2004 and 2014 **eFigure 4.** Evolution of the Kidney Donor Risk Profile (KDPI) in the US and in France over time **eFigure 5.** ROC curve representing the probability of deceased donor discard according to the Kidney Donor Risk Index in the US and in France and the related calibration curves **eFigure 6.** Kaplan-Meier curves for death-censored kidney-graft survival, according to the deciles of the Kidney Donor Risk Index (KDRI) in the US **eFigure 7.** Restricted mean survival time at 10 years in the US for the 10 deciles of KDRI **eFigure 8.** Kaplan-Meier curves for kidney-graft survival, according to the deciles of the Kidney Donor Risk Index in France **eFigure 9.** Kaplan-Meier curves for non-death-censored kidney-graft survival, according to the deciles of the Kidney Donor Risk Index in the US after transplantation **eFigure 10.** Kaplan-Meier curves for non-death-censored kidney-graft survival, according to the deciles of the Kidney Donor Risk Index in France after transplantation **eFigure 11.** Estimation of non-death-censored lost allograft life years from high kidney discard rates in the US, by KDRI categories

## References

[1] Axelrod DA, Schnitzler MA, Xiao H, An economic assessment of contemporary kidney transplant practice. Am J Transplant. 2018;18(5):1168-1176. doi:10.1111/ajt.14702 29451350

[2] Eurotransplant. https://www.eurotransplant.org/cms/. Accessed January 7, 2019.

[3] Hart A, Smith JM, Skeans MA, OPTN/SRTR 2015 Annual Data Report: Kidney. Am J Transplant. 2017;17(suppl 1):21-116. doi:10.1111/ajt.14124 28052609 PMC5527691

[4] Organ Procurement and Transplantation Network (OPTN). https://optn.transplant.hrsa.gov/. Accessed January 7, 2019.

[5] Reese PP, Harhay MN, Abt PL, Levine MH, Halpern SD New solutions to reduce discard of kidneys donated for transplantation. J Am Soc Nephrol. 2016;27(4):973-980. doi:10.1681/ASN.2015010023 26369343 PMC4814180

[6] Stewart DE, Garcia VC, Rosendale JD, Klassen DK, Carrico BJ Diagnosing the decades-long rise in the deceased donor kidney discard rate in the United States. Transplantation. 2017;101(3):575-587. doi:10.1097/TP.0000000000001539 27764031

[7] Gill J, Joffres Y, Rose C, The change in living kidney donation in women and men in the United States (2005-2015): a population-based analysis. J Am Soc Nephrol. 2018;29(4):1301-1308. doi:10.1681/ASN.2017111160 29519800 PMC5875963

[8] Tullius SG, Rabb H Improving the supply and quality of deceased-donor organs for transplantation. N Engl J Med. 2018;378(20):1920-1929. doi:10.1056/NEJMra1507080 29768153

[9] Massie AB, Luo X, Chow EK, Alejo JL, Desai NM, Segev DL Survival benefit of primary deceased donor transplantation with high-KDPI kidneys. Am J Transplant. 2014;14(10):2310-2316. doi:10.1111/ajt.12830 25139729

[10] Merion RM, Ashby VB, Wolfe RA, Deceased-donor characteristics and the survival benefit of kidney transplantation. JAMA. 2005;294(21):2726-2733. doi:10.1001/jama.294.21.2726 16333008

[11] Cohen JB, Eddinger KC, Locke JE, Forde KA, Reese PP, Sawinski DL Survival benefit of transplantation with a deceased diabetic donor kidney compared with remaining on the waitlist. Clin J Am Soc Nephrol. 2017;12(6):974-982. doi:10.2215/CJN.10280916 28546439 PMC5460711

[12] Schold JD, Buccini LD, Srinivas TR, The association of center performance evaluations and kidney transplant volume in the United States. Am J Transplant. 2013;13(1):67-75. doi:10.1111/j.1600-6143.2012.04345.x 23279681

[13] Whiting JF, Golconda M, Smith R, O’Brien S, First MR, Alexander JW Economic costs of expanded criteria donors in renal transplantation. Transplantation. 1998;65(2):204-207. doi:10.1097/00007890-199801270-00010 9458015

[14] Saidi RF, Elias N, Kawai T, Outcome of kidney transplantation using expanded criteria donors and donation after cardiac death kidneys: realities and costs. Am J Transplant. 2007;7(12):2769-2774. doi:10.1111/j.1600-6143.2007.01993.x 17927805

[15] Kasiske BL, Stewart DE, Bista BR, The role of procurement biopsies in acceptance decisions for kidneys retrieved for transplant. Clin J Am Soc Nephrol. 2014;9(3):562-571. doi:10.2215/CJN.07610713 24558053 PMC3944771

[16] Ekser B, Powelson JA, Fridell JA, Goggins WC, Taber TE Is the kidney donor profile index (KDPI) universal or UNOS-specific? Am J Transplant. 2018;18(4):1031-1032. 29024392 10.1111/ajt.14538

[17] Rao PS, Schaubel DE, Guidinger MK, A comprehensive risk quantification score for deceased donor kidneys: the kidney donor risk index. Transplantation. 2009;88(2):231-236. doi:10.1097/TP.0b013e3181ac620b 19623019

[18] Bae S, Massie AB, Luo X, Anjum S, Desai NM, Segev DL Changes in discard rate after the introduction of the Kidney Donor Profile Index (KDPI). Am J Transplant. 2016;16(7):2202-2207. doi:10.1111/ajt.13769 26932575 PMC4925251

[19] Frei U, Noeldeke J, Machold-Fabrizii V, Prospective age-matching in elderly kidney transplant recipients—a 5-year analysis of the Eurotransplant Senior Program. Am J Transplant. 2008;8(1):50-57. 17973969 10.1111/j.1600-6143.2007.02014.x

[20] Rose C, Schaeffner E, Frei U, Gill J, Gill JS A lifetime of allograft function with kidneys from older donors. J Am Soc Nephrol. 2015;26(10):2483-2493. doi:10.1681/ASN.2014080771 25814474 PMC4587698

[21] Cristal Agence de la Biomédecine http://www.sipg.sante.fr/portail/. Accessed January 7, 2019.

[22] Strang WN, Tuppin P, Atinault A, Jacquelinet C The French organ transplant data system. Stud Health Technol Inform. 2005;116:77-82. 16160239

[23] Agence de la Biomédecine https://www.agence-biomedecine.fr/. Accessed January 7, 2019.

[24] Lehner LJ, Kleinsteuber A, Halleck F, Assessment of the Kidney Donor Profile Index in a European cohort. Nephrol Dial Transplant. 2018;33(8):1465-1472. doi:10.1093/ndt/gfy030 29617898

[25] Peters-Sengers H, Heemskerk MBA, Geskus RB, Validation of the Prognostic Kidney Donor Risk Index Scoring System of Deceased Donors for Renal Transplantation in the Netherlands. Transplantation. 2018;102(1):162-170. doi:10.1097/TP.0000000000001889 28731905

[26] Metropolis N The beginning of the Monte Carlo method. Stanislaw Ulam Los Alamos Sci. 1987;15:125-130.

[27] Efron B Nonparametric estimates of standard error: The jackknife, the bootstrap and other methods. Biometrika. 1981;63:589-599. doi:10.1093/biomet/68.3.589

[28] Lamb KE, Lodhi S, Meier-Kriesche HU Long-term renal allograft survival in the United States: a critical reappraisal. Am J Transplant. 2011;11(3):450-462. doi:10.1111/j.1600-6143.2010.03283.x 20973913

[29] Mohan S, Chiles MC, Patzer RE, Factors leading to the discard of deceased donor kidneys in the United States. Kidney Int. 2018;94(1):187-198. doi:10.1016/j.kint.2018.02.016 29735310 PMC6015528

[30] Young EW, Goodkin DA, Mapes DL, The Dialysis Outcomes and Practice Patterns Study (DOPPS): An international hemodialysis study. Kidney Int. 2000;57(suppl 74):S-74-S-81. doi:10.1046/j.1523-1755.2000.07413.x

[31] Rosenblum AM, Li AH, Roels L, Worldwide variability in deceased organ donation registries. Transpl Int. 2012;25(8):801-811. doi:10.1111/j.1432-2277.2012.01472.x 22507140 PMC3440579

[32] Shepherd L, O’Carroll RE, Ferguson E An international comparison of deceased and living organ donation/transplant rates in opt-in and opt-out systems: a panel study. BMC Med. 2014;12:131. doi:10.1186/s12916-014-0131-4 25285666 PMC4175622

[33] Rao PS, Merion RM, Ashby VB, Port FK, Wolfe RA, Kayler LK Renal transplantation in elderly patients older than 70 years of age: results from the Scientific Registry of Transplant Recipients. Transplantation. 2007;83(8):1069-1074. doi:10.1097/01.tp.0000259621.56861.31 17452897

[34] Schold JD, Meier-Kriesche HU Which renal transplant candidates should accept marginal kidneys in exchange for a shorter waiting time on dialysis? Clin J Am Soc Nephrol. 2006;1(3):532-538. doi:10.2215/CJN.01130905 17699256

[35] Peters-Sengers H, Berger SP, Heemskerk MB, Stretching the limits of renal transplantation in elderly recipients of grafts from elderly deceased donors. J Am Soc Nephrol. 2017;28(2):621-631. doi:10.1681/ASN.2015080879 27729570 PMC5280003

[36] Pérez-Sáez MJ, Arcos E, Comas J, Crespo M, Lloveras J, Pascual J; Catalan Renal Registry Committee Survival benefit from kidney transplantation using kidneys from deceased donors aged ≥75 years: a time-dependent analysis. Am J Transplant. 2016;16(9):2724-2733. doi:10.1111/ajt.13800 27004984

[37] Collini A, Kalmar P, Dhamo A, Ruggieri G, Carmellini M Renal transplant from very old donors: how far can we go? Transplantation. 2009;87(12):1830-1836. doi:10.1097/TP.0b013e3181a6b4ff 19543060

[38] Goldberg DS, Halpern SD, Reese PP Deceased organ donation consent rates among racial and ethnic minorities and older potential donors. Crit Care Med. 2013;41(2):496-505. doi:10.1097/CCM.0b013e318271198c 23263585 PMC3557536

[39] Gill JS, Tonelli M Penny wise, pound foolish? Coverage limits on immunosuppression after kidney transplantation. N Engl J Med. 2012;366(7):586-589. doi:10.1056/NEJMp1114394 22296029

[40] Merion RM, Goodrich NP, Johnson RJ, Kidney transplant graft outcomes in 379 257 recipients on 3 continents. Am J Transplant. 2018;18(8):1914-1923. doi:10.1111/ajt.14694 29573328

[41] Ojo AO, Morales JM, González-Molina M, ; Scientific Registry of Transplant Recipients and; Spanish Chronic Allograft Study Group Comparison of the long-term outcomes of kidney transplantation: USA versus Spain. Nephrol Dial Transplant. 2013;28(1):213-220. doi:10.1093/ndt/gfs287 22759384 PMC3616762

[42] Tullius SG, Milford E Kidney allocation and the aging immune response. N Engl J Med. 2011;364(14):1369-1370. doi:10.1056/NEJMc1103007 21410395

