# Supplementary material for: Disparities in Acceptance of Deceased Donor Kidneys Between the United States and France and Estimated Effects of Increased US Acceptance
Source: JAMA Intern Med. 2019 Aug 26;179(10):1365–74. doi: 10.1001/jamainternmed.2019.2322 (PMC6714020; doi:10.1001/jamainternmed.2019.2322)
Supplement: Supplement 2. — eMethods eTable 1A. US cohort: Donor characteristics associated with kidney discard bivariate analysis eTable 1B. US cohort: Donor characteristics associated with kidney discard multivariable analysis eTable 2A. French Cohort: Donor characteristics associated with kidney discard bivariate analysis eTable 2B. French Cohort: Donor characteristics associated with kidney discard in France in multivariable analysis eFigure 1. Flowchart of the study population in the US and in France eFigure 2. Distribution of deceased donor kidney quality as manifested in the Kidney Donor Risk Profile (KDPI) kidneys in the US and in France eFigure 3. Change in the mean deceased donor age and Kidney Donor Risk Index in the US and France between 2004 and 2014 eFigure 4. Evolution of the Kidney Donor Risk Profile (KDPI) in the US and in France over time eFigure 5. ROC curve representing the probability of deceased donor discard according to the Kidney Donor Risk Index in the US and in France and the related calibration curves eFigure 6. Kaplan-Meier curves for death-censored kidney-graft survival, according to the deciles of the Kidney Donor Risk Index (KDRI) in the US eFigure 7. Restricted mean survival time at 10 years in the US for the 10 deciles of KDRI eFigure 8. Kaplan-Meier curves for kidney-graft survival, according to the deciles of the Kidney Donor Risk Index in France eFigure 9. Kaplan-Meier curves for non-death-censored kidney-graft survival, according to the deciles of the Kidney Donor Risk Index in the US after transplantation eFigure 10. Kaplan-Meier curves for non-death-censored kidney-graft survival, according to the deciles of the Kidney Donor Risk Index in France after transplantation eFigure 11. Estimation of non-death-censored lost allograft life years from high kidney discard rates in the US, by KDRI categories [file jamainternmed-179-1365-s002.pdf]

Supplementary Online Content

Aubert O, Reese PP, Audry B, et al. Disparities in acceptance of deceased donor kidneys between the United States and France and estimated effects of increased US acceptance. Published online August 26, 2019. *JAMA Intern Med*. doi:10.1001/jamainternmed.2019.2322

eMethods
eTable 1A. US cohort: Donor characteristics associated with kidney discard bivariate analysis
eTable 1B. US cohort: Donor characteristics associated with kidney discard multivariable analysis
eTable 2A. French Cohort: Donor characteristics associated with kidney discard bivariate analysis
eTable 2B. French Cohort: Donor characteristics associated with kidney discard in France in multivariable analysis
eFigure 1. Flowchart of the study population in the US and in France
eFigure 2. Distribution of deceased donor kidney quality as manifested in the Kidney Donor Risk Profile (KDPI) kidneys in the US and in France
eFigure 3. Change in the mean deceased donor age and Kidney Donor Risk Index in the US and France between 2004 and 2014
eFigure 4. Evolution of the Kidney Donor Risk Profile (KDPI) in the US and in France over time
eFigure 5. ROC curve representing the probability of deceased donor discard according to the Kidney Donor Risk Index in the US and in France and the related calibration curves
eFigure 6. Kaplan-Meier curves for death-censored kidney-graft survival, according to the deciles of the Kidney Donor Risk Index (KDRI) in the US
eFigure 7. Restricted mean survival time at 10 years in the US for the 10 deciles of KDRI
eFigure 8. Kaplan-Meier curves for kidney-graft survival, according to the deciles of the Kidney Donor Risk Index in France
eFigure 9. Kaplan-Meier curves for non-death-censored kidney-graft survival, according to the deciles of the Kidney Donor Risk Index in the US after transplantation
eFigure 10. Kaplan-Meier curves for non-death-censored kidney-graft survival, according to the deciles of the Kidney Donor Risk Index in France after transplantation
eFigure 11. Estimation of non-death-censored lost allograft life years from high kidney discard rates in the US, by KDRI categories

This supplementary material has been provided by the authors to give readers additional information about their work.

© 2019 Olivier A et al. *JAMA Internal Medicine*.

© 2019 Olivier A et al. *JAMA Internal Medicine*.

1. eMETHODS

1.1. Interpretation of statistical analyses

1.1.1. Discrimination

The aim of discrimination is to distinguish between patients who experience an event from those who do not. The AUC (0≤C≤1) is the probability of concordance between predicted and observed events, with AUC=0.5 for random predictions and AUC=1 for a perfectly discriminating model.

1.1.2. Calibration

Calibration refers to the ability to provide unbiased predictions in groups of similar patients. It estimates how close the estimated risk is to the observed risk. A prediction model is considered “well-calibrated” if the difference between predictions and observations in all groups of similar patients is close to 0 (perfect calibration). Any large deviation (P<0.1) indicates a lack of calibration.

1.1.3. Monte-Carlo technique

Acceptance practices were simulated for each kidney separately (not at the donor level). For each kidney *k* of a given deceased donor *d* from one of our 2 countries, the following procedures were performed: (i) a uniform 0-to-1 random number U_{dk} was generated; and (ii) its probability P_{dk} of being discarded was computed from the other-country logistic regression model according to the actual KDRI K_d of the donor, and the kidney *k* was virtually discarded if and only if $U_{dk} \leq P_{dk}$.

1.2. Calculation of the Kidney Donor Risk Index (KDRI) and the Kidney Donor Profile Index (KDPI)

The KDRI and KDPI are scores derived from 10 deceased donor variables and predict risk of kidney allograft failure after transplantation. Lower values for the KDRI and KDPI indicate kidneys with better projected allograft survival. These indices were developed for the purpose of enabling clinicians to try to make rough assessments of allograft quality and graft failure risk across donors with different attributes.(1) A guidance document from the United Network for Organ Sharing states “The Kidney Donor Profile Index (KDPI) is a numerical measure that combines ten donor

© 2019 Olivier A et al. *JAMA Internal Medicine*.

factors, including clinical parameters and demographics, to summarize into a single number the quality of deceased donor kidneys relative to other recovered kidneys.” (2)

The KDRI score for any kidney allograft estimates the risk of failure for that allograft compared to a kidney from a reference donor. This reference donor is defined as 40 years old, non-African-American, 170 cm tall, weighing 80 kg, with a creatinine level of 1 mg/dL, as well as negative history of hypertension, diabetes and hepatitis C virus serostatus. Notably, race/ethnicity for organ donors is not available according to French national bioethics rules. As a result, as we entered “non-black” as the race for all French donors when calculating KDRI. This approach to calculating the KDRI of the French pool of donated kidneys will have the net effect of slightly over-estimating the quality of these organs. Nonetheless, as shown in the results, French transplant centers are still much more likely to accept kidneys with the highest KDRI scores (i.e. lowest quality kidneys) compared to US centers.

By convention, we mapped the calculated KDRI values onto a cumulative percentage scale from 0-100 to generate the KDPI. Because our analysis focused on kidneys recovered from 2004 to 2014, as recommended by the OPTN, the 2015 scaling factor for converting “KDRI Rao” to the KDRI median was used. Lower values for the KDRI indicate kidneys with better projected allograft survival. The KDRI and KDPI are not formally used in kidney allocation in France, but donor characteristics used to calculate the scores (with the exception of donor race) are presented to transplant centers with organ offers.

1.3. Systematic literature review supporting the study novelty

We conducted a comprehensive search of PubMed Plus and EBSCO MegaFILE using the keywords “kidney transplantation” and “discard” for all articles published from October 2008 to March 2018. Search: ((("kidney transplantation") AND discard) AND ("2008/10"[Date - Publication]: "3000"[Date - Publication])). Two investigators independently reviewed the results and eliminated four irrelevant publications, leaving 104 publications. We concluded that similar deceased donor characteristics – such as older age or acute kidney injury – elevate the risk of deceased donor kidney discard in transplant systems across multiple countries. Finally, the systematic review revealed that no studies systematically assessed kidney discard rates between different countries and their impact in term of gain or loss of opportunities for waitlisted patients. No study has used computer simulation algorithms to address the possibility of virtually applying a European Union-based approach of kidney allocation and acceptance to the US transplant system.

© 2019 Olivier A et al. *JAMA Internal Medicine*.

2. SUPPLEMENTARY TABLES

eTable 1A, 1B, 2A and 2B: Estimation of the number of donated kidneys that would be discarded in the US and in France using prediction models integrating donor variables instead the single KDRI score. The AUC of the final model was 0.821 (95% CI [0.818-0.824]) and 0.744 (95% CI [0.734-0.754]) for the US and French based models, respectively, showing similar results to the original model that included only the single KDRI score as a covariate. Applying the French model using donor variables instead of the single KDRI score to the US cohort still resulted in a major decrease in the discarded kidney rate from n=27,987/156,089 (17.9%) to n=14,884/156,089 (9.5%). Moreover, applying the US model using all variables instead of the single KDRI score to the French cohort still resulted in a major increase in the discarded kidney rate (n=6,687/29,984 = 22.3%). Applying the French model using donor variables instead of the single KDRI score to the US cohort still resulted in a major decrease in the discarded kidneyrate from n=27,987/156,089 (17.9%) to n=14,884/156,089 (9.5%).

© 2019 Olivier A et al. *JAMA Internal Medicine*.

eTable 1A: US cohort: Donor characteristics associated with kidney discard in bivariate analysis

			Number of patients	Number of discards	OR	95% CI	p
**Donor characteristics**	**Donor age (**per 1-year increment)		156,089	27,987	1.062	(1.061 - 1.063)	**<0.0001**
	**Donor sex**	**Female**	63,700	13,293	1	-	
		**Male**	92,389	14,694	0.717	(0.699 – 0.736)	**<0.0001**
	**BMI** (per 1 kg/m^2 increment)		156,009	27,987	1.037	(1.035 – 1.038)	**<0.0001**
	**Donation after cardiac death (DCD) organ recovery**	**No**	137,065	24,171	1	-	
		**Yes**	18,853	3,816	1.187	(1.143 – 1.233)	**<0.0001**
	**Cerebrovascular accident as cause of death**	**No**	98,125	11,881	1	-	
		**Yes**	57,964	16,106	2.793	(2.720 – 2.868)	**<0.0001**
	**Donor hypertension**	**No**	107,568	11,186	1	-	
		**Yes**	48,521	16,801	4.534	(4.442 – 4.689)	**<0.0001**
	**Donor diabetes mellitus**	**No**	141,801	21,784	1	-	
		**Yes**	14,288	6,203	4.227	(4.077 – 4.382)	**<0.0001**
	**Creatinine**	**<1.5 mg/dL**	124,268	17,547	1	-	
		**≥1.5 mg/dL**	31,821	10,440	2.970	(2.887 – 3.055)	**<0.0001**
	**Hepatitis C virus serostatus**	**Negative**	150,284	25,018	1	-	
		**Positive**	5,644	2,932	5.413	(5.129 – 5.713)	**<0.0001**
	**African American donor**	**No**	133,338	23,239	1	-	
		**Yes**	27,751	4,748	1.249	(1.207 – 1.294)	**<0.0001**

eTable 1B: US cohort : Donor characteristics associated with kidney discard in multivariable analysis

			Number of patients	Number of discards	OR	95% CI	p
**Donor characteristics**	**Donor age (**per 1-year increment)		155,767	27,986	1.053	(1.052 - 1.055)	**<0.0001**
	**Donor gender**	**Female**	63,553	13,250	1	-	
		**Male**	92,214	14,646	0.750	(0.727 – 0.773)	**<0.0001**
	**BMI** (per 1 kg/m^2 increment)		155,767	27,896	0.982	(0.979 – 0.984)	**<0.0001**
	**Donation after cardiac death (DCD) organ recovery**	**No**	136,975	24,107	1	-	
		**Yes**	17,792	3,789	1.950	(1.866 – 2.038)	**<0.0001**
	**Cerebrovascular accident as cause of death**	**No**	97,937	11,846	1	-	
		**Yes**	57,830	16,050	1.166	(1.128 – 1.206)	**<0.0001**
	**Donor hypertension**	**No**	107,312	11,135	1	-	
		**Yes**	48,455	16,761	1.846	(1.784 – 1.910)	**<0.0001**
	**Donor diabetes mellitus**	**No**	141,503	21,702	1	-	
		**Yes**	14,264	6,194	2.143	(2.055 – 2.236)	**<0.0001**
	**Creatinine**	**<1.5 mg/dL**	123,992	17,479	1	-	
		**≥1.5 mg/dL**	31,775	10,417	3.622	(3.501 – 3.747)	**<0.0001**
	**Hepatitis C virus serostatus**	**Negative**	150,129	24,968	1	-	
		**Positive**	5,638	2,928	7.864	(7.398 – 8.359)	**<0.0001**
	**African American donor**	**No**	133,076	23,168	1	-	
		**Yes**	22,691	4,728	1.078	(1.034 – 1.125)	**<0.0001**

© 2019 Olivier A et al. *JAMA Internal Medicine*.

eTable 2A: French Cohort: Donor characteristics associated with kidney discard in bivariate analysis

			Number of patients	Number of discards	OR	95% CI	p
**Donor characteristics**	**Donor age (**per 1-year increment)		29,984	2,732	1.042	(1.039 - 1.045)	**<0.0001**
	**Donor gender**	**Female**	12,164	1,022	1	-	
		**Male**	17,820	1,710	1,157	(1.067 – 1.255)	**0.0004**
	**BMI** (per 1 kg/m^2 increment)		29,984	2,732	1.040	(1.032 – 1.047)	**<0.0001**
	**Donation after cardiac death (DCD) organ recovery**	**No**	29,347	2,533	1	-	
		**Yes**	637	199	4.810	(4.048 – 5.715)	**<0.0001**
	**Cerebrovascular accident as cause of death**	**No**	13,297	916	1	-	
		**Yes**	16,687	1,816	1.650	(1.519 – 1.793)	**<0.0001**
	**Donor hypertension**	**No**	20,726	1,383	1	-	
		**Yes**	9,268	1,349	2.381	(2.199 – 2.578)	**<0.0001**
	**Donor diabetes mellitus**	**No**	27,761	2,353	1	-	
		**Yes**	2,223	379	2.279	(1.972 – 2.498)	**<0.0001**
	**Creatinine**	**<1.5 mg/dL**	25,936	2,104	1	-	
		**≥1.5 mg/dL**	4,048	628	2.080	(1.889 – 2.290)	**<0.0001**
	**Hepatitis C virus serostatus**	**Negative**	29,919	2,703	1	-	
		**Positive**	65	29	8.111	(4.966 – 13.248)	**<0.0001**

eTable 2B: French Cohort: Donor characteristics associated with kidney discard in France in multivariable analysis

			Number of patients	Number of discards	OR	95% CI	p
**Donor characteristics**	**Donor age (**per 1-year increment)		29,984	2,732	1.043	(1.040 - 1.047)	**<0.0001**
	**Donor gender**	**Female**	12,164	1,022	1	-	
		**Male**	17,820	1,710	1.262	(1.156 – 1.378)	**<0.0001**
	**BMI** (per 1 kg/m^2 increment)		29,984	2,732	0.994	(0.995 – 1.002)	**0.145**
	**Donation after cardiac death (DCD) organ recovery**	**No**	29,347	2,533	1	-	
		**Yes**	637	199	8.537	(7.009 – 10.399)	**<0.0001**
	**Cerebrovascular accident as cause of death**	**No**	13,297	916	1	-	
		**Yes**	16,687	1,816	1.283	(1.164 – 1.415)	**<0.0001**
	**Donor hypertension**	**No**	20,726	1,383	1	-	
		**Yes**	9,268	1,349	1.460	(1.329 – 1.604)	**<0.0001**
	**Donor diabetes mellitus**	**No**	27,761	2,353	1	-	
		**Yes**	2,223	379	1.444	(1.271 – 1.640)	**<0.0001**
	**Creatinine**	**<1.5 mg/dL**	25,936	2,104	1	-	
		**≥1.5 mg/dL**	4,048	628	2.063	(1.852 – 2.296)	**<0.0001**
	**Hepatitis C virus serostatus**	**Negative**	29,919	2,703	1	-	
		**Positive**	65	29	12.527	(7.334 – 21.398)	**<0.0001**

** Note that reporting of donor ethnicity is not allowed in the French database system according to national regulations and was not considered in this analysis.*

© 2019 Olivier A et al. *JAMA Internal Medicine*.

3. SUPPLEMETARY FIGURES

eFigure 1A: Flowchart of the study population in the US: 157,614 deceased donor kidneys recovered for transplant between 2004 and 2014.

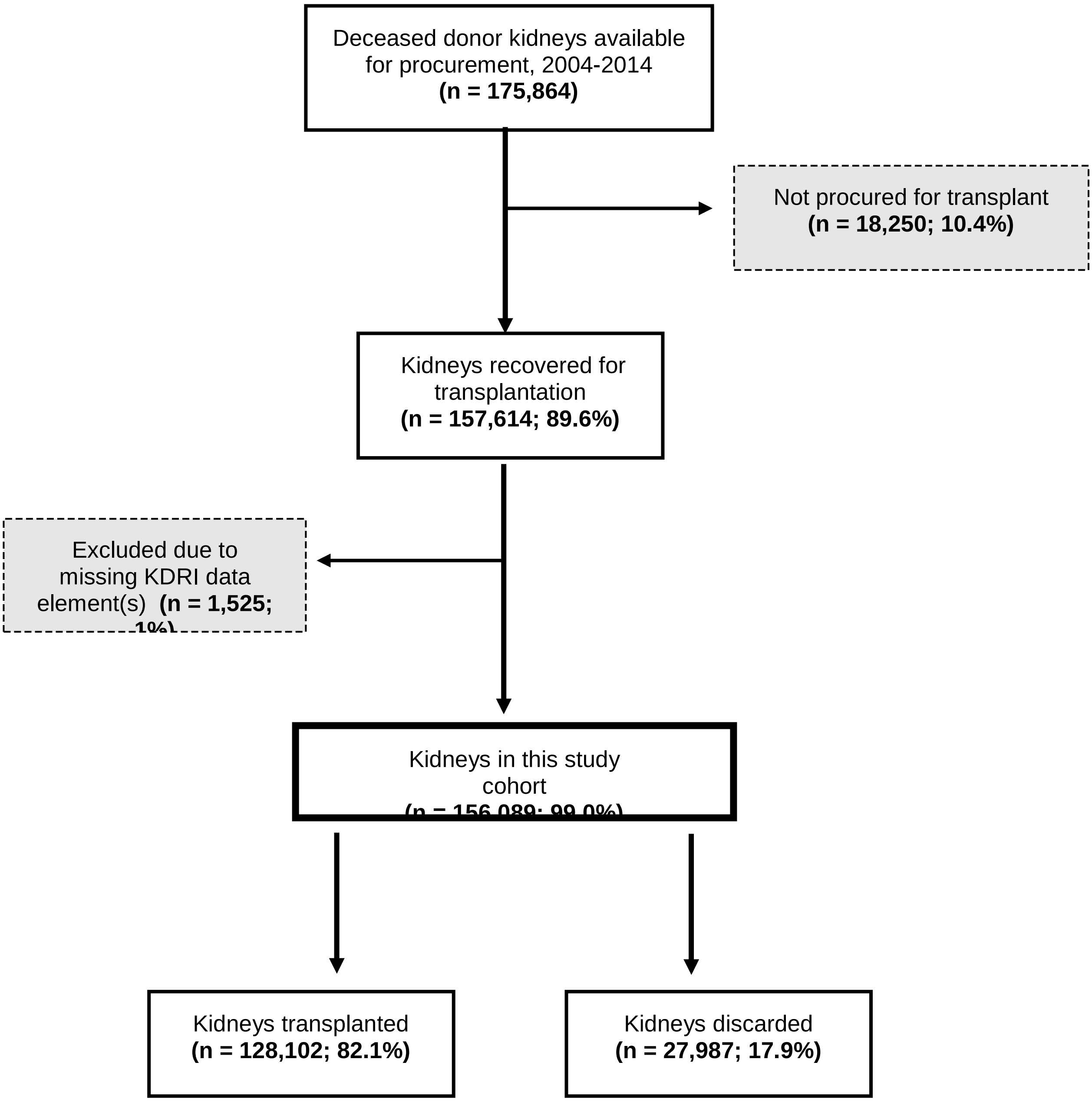

KDRI: Kidney Donor Risk Index.

© 2019 Olivier A et al. *JAMA Internal Medicine*.

eFigure 1B: Flowchart of the study population in France: 31,387 deceased donor kidneys recovered for transplant between 2004 and 2014.

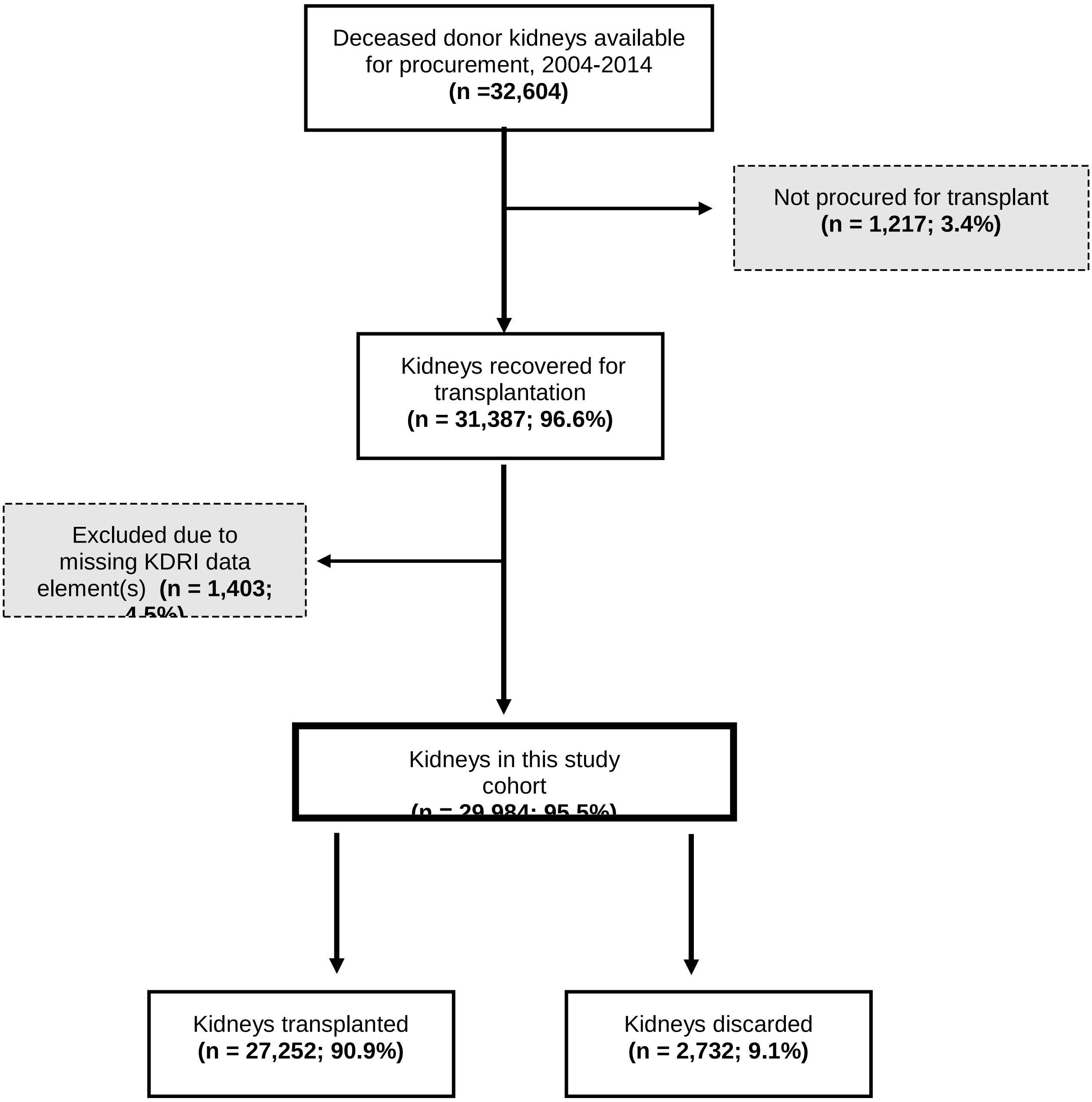

KDRI: Kidney Donor Risk Index.

© 2019 Olivier A et al. *JAMA Internal Medicine*.

eFigure 2: Distribution of deceased donor kidney quality as manifested in the Kidney Donor Risk Profile (KDPI) kidneys in the US and in France. Panel A shows the distribution of the KDPI score for the transplanted (blue) and discarded kidneys (red) in the US. Panel B shows the distribution of the KDPI score for the transplanted (blue) and discarded kidneys (red) in France. For instance, for a KDPI score of 100%, 78% of the kidneys were discarded in the US, while 29% of the kidneys were discarded in France.

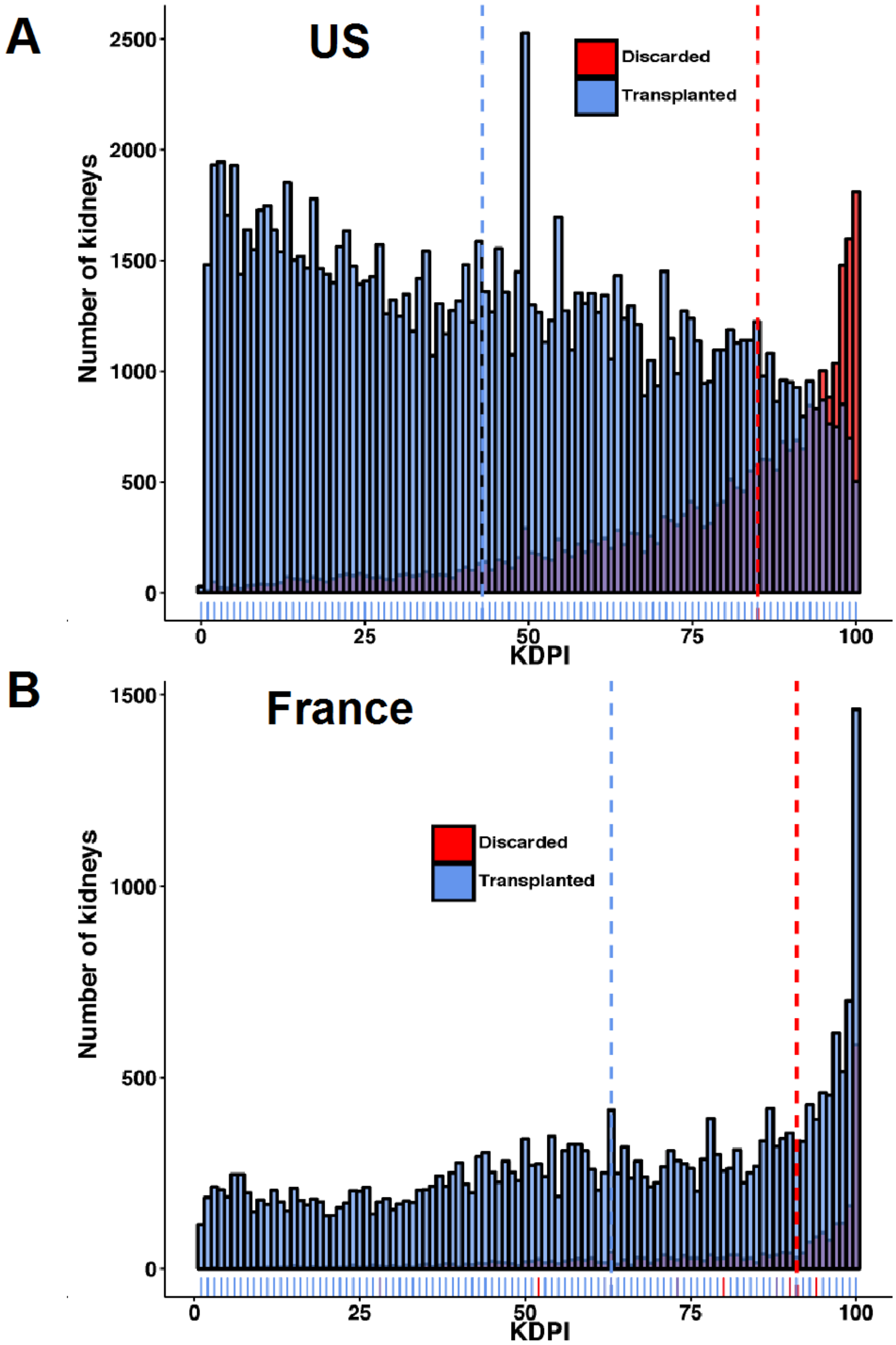

Abbreviations: KDPI, Kidney Donor Risk Profile.

© 2019 Olivier A et al. *JAMA Internal Medicine*.

eFigure 3: Change in the mean deceased donor age and Kidney Donor Risk Index (KDRI) in the US and France between 2004 and 2014.

Panel A shows the mean donor age over time in the US for transplanted (black) and discarded (blue) kidneys. Panel B shows the mean donor age over time in France for transplanted (black) and discarded (blue) kidneys. Panel C shows the mean KDRI over time in the US for transplanted (black) and discarded (blue) kidneys. Panel D shows the mean KDRI over time in France for transplanted (black) and discarded (blue) kidneys.

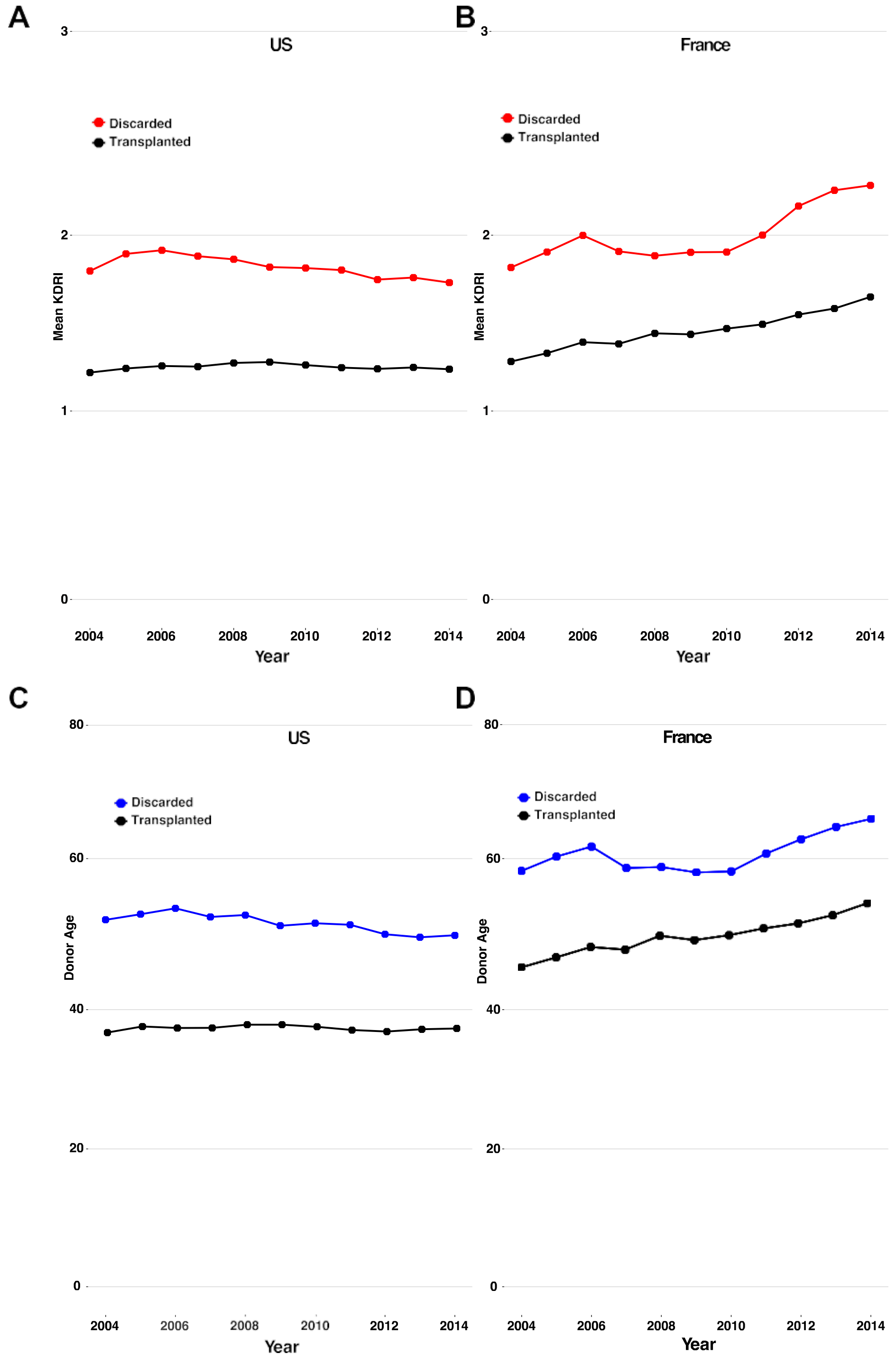

Abbreviations: KDRI; Kidney Donor Risk Index.

© 2019 Olivier A et al. *JAMA Internal Medicine*.

eFigure 4: Evolution of the Kidney Donor Risk Profile (KDPI) in the US and in France over time. This shows the evaluation of the KDPI over time in the USA and in France for the transplanted (gray) and discarded (red) kidneys. The mean KDPI of transplanted kidneys was 43%±28% in 2004 and 45%±27% in 2014 in the US, while it was 55%±29% in 2004 and 69%±30% in 2014.

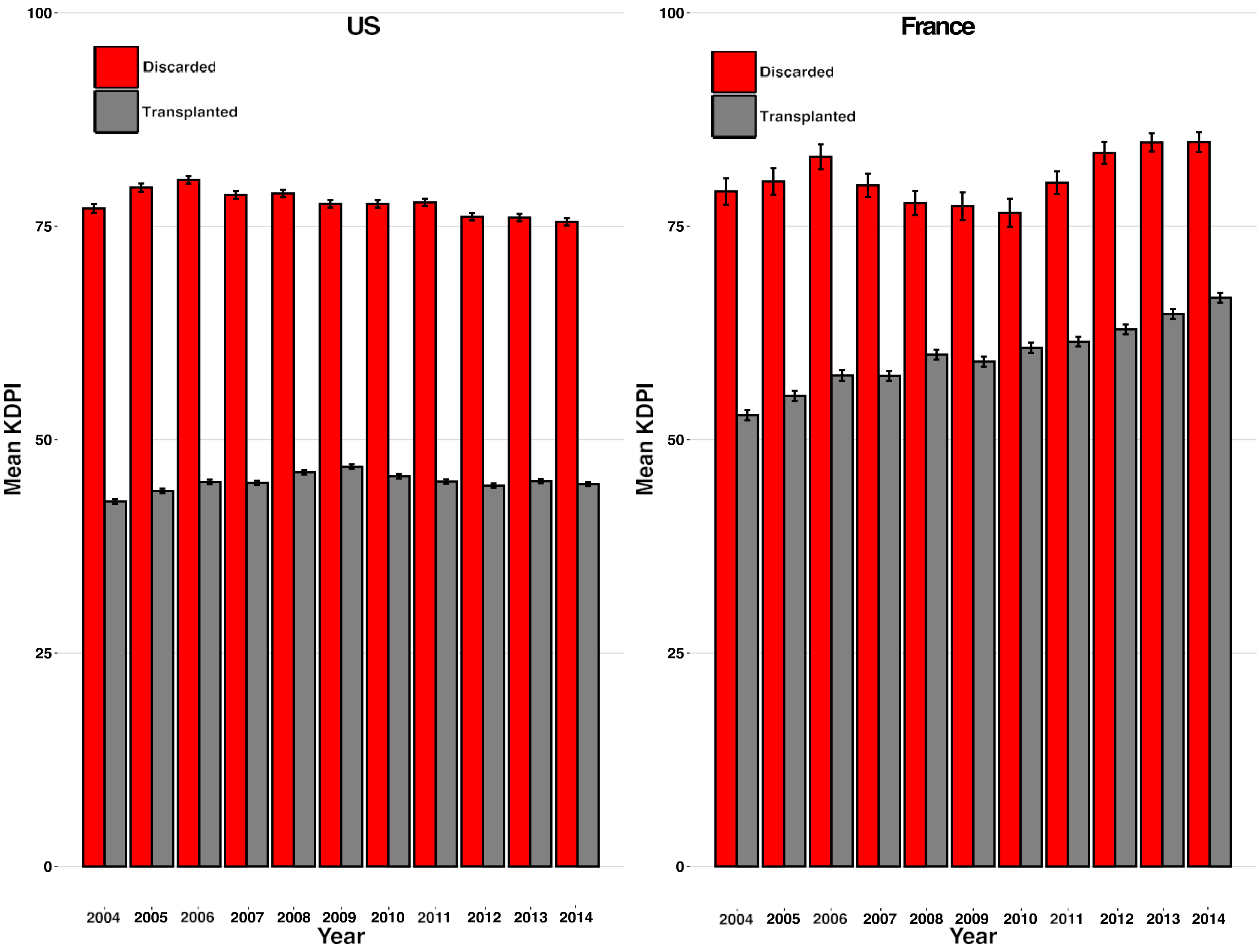

Abbreviations: KDPI; Kidney Donor Risk Index.

© 2019 Olivier A et al. *JAMA Internal Medicine*.

eFigure 5: ROC curve representing the probability of deceased donor discard according to the Kidney Donor Risk Index in the US and in France and the related calibration curves.

Panel A shows the ROC curves of the KDRI in the US (red; AUC=0.82) and in France (blue; AUC=0.72). Panels B and C show the calibrations of the KDRI model in the US and in France. The vertical axis is the observed proportion of grafts discarded. The x-axis shows the probability of discard using the model. The dashed black lines correspond to the ideal calibration, the red curve corresponds to the bias-corrected calibration in the US, and the blue curve corresponds to the bias-corrected calibration in France.

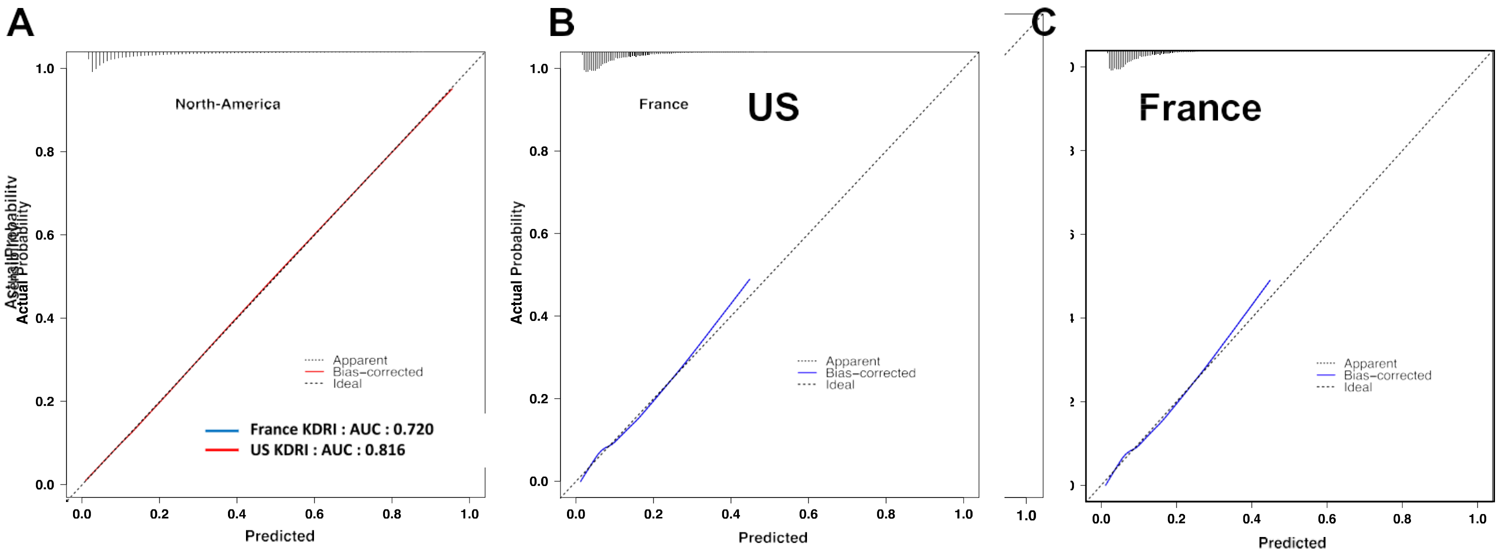

© 2019 Olivier A et al. *JAMA Internal Medicine*.

eFigure 6: Kaplan-Meier curves for death-censored kidney-graft survival, according to the deciles of the Kidney Donor Risk Index (KDRI) in the US.

This figure shows the probability of graft survival among actual kidney transplant recipients in the US and stratified by the KDRI deciles. The black curve corresponds to KDRI decile 1, the grey curve to KDRI decile 2, the green curve to KDRI decile 3, the light blue curve to KDRI decile 4, the dark blue curve to KDRI decile 5, the yellow curve to KDRI decile 6, the orange curve to KDRI decile 7, the pink curve to KDRI decile 8, the purple curve to KDRI decile 9, and the red curve to KDRI decile 10. The overall difference was significantly different (log-rank p<0.0001).

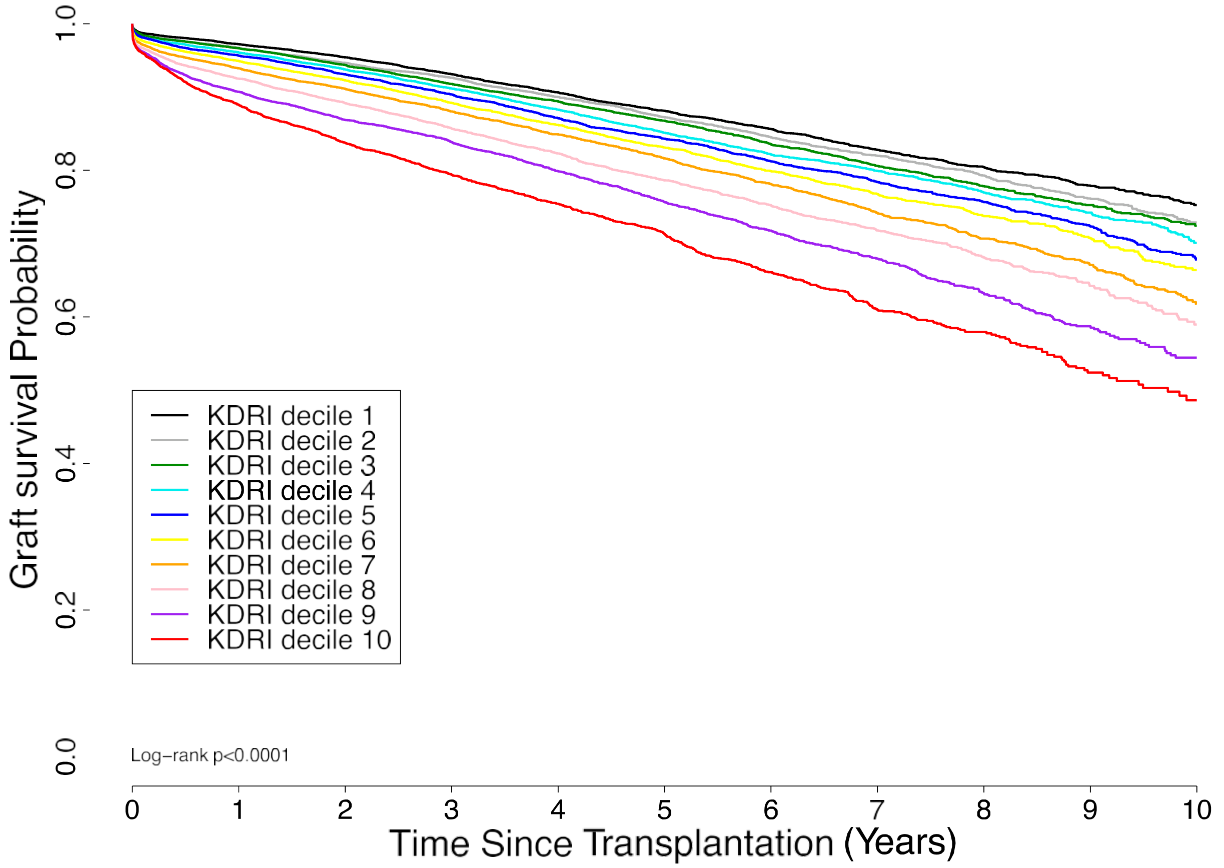

N at Risk	0	1	2	3	4	5	6	7	8	9	10
KDR decile 1	15352	12703	10760	8854	7178	5651	4342	3192	2120	1182	396
KDRI decile 2	15089	12333	10382	8524	6724	5226	3893	2850	1820	985	327
KDRI decile 3	14859	12068	9977	8088	6414	4962	3695	2600	1659	880	307
KDRI decile 4	14634	11717	9640	7836	6114	4682	3412	2408	1504	819	256
KDRI decile 5	14082	11274	9211	7433	5872	4483	3267	2248	1460	782	274
KDRI decile 6	13194	10466	8606	6914	5425	4094	2932	2003	1211	610	218
KDRI decile 7	12215	9534	7752	6195	4822	3671	2641	1819	1120	601	218
KDRI decile 8	10863	8340	6719	5351	4149	3058	2187	1464	867	412	126
KDRI decile 9	9063	6865	5493	4391	3329	2425	1681	1046	634	311	97
KDRI decile 10	5445	4040	3244	2561	1956	1414	950	606	351	160	48

Abbreviations: KDRI; Kidney Donor Risk Index.

© 2019 Olivier A et al. *JAMA Internal Medicine*.

eFigure 7: Restricted mean survival time at 10 years in the US for the 10 deciles of the KDRI. The pink area under the curve corresponds to the mean survival probability at ten years. The orange area above the curve corresponds to the mean years of allograft lost at ten years.

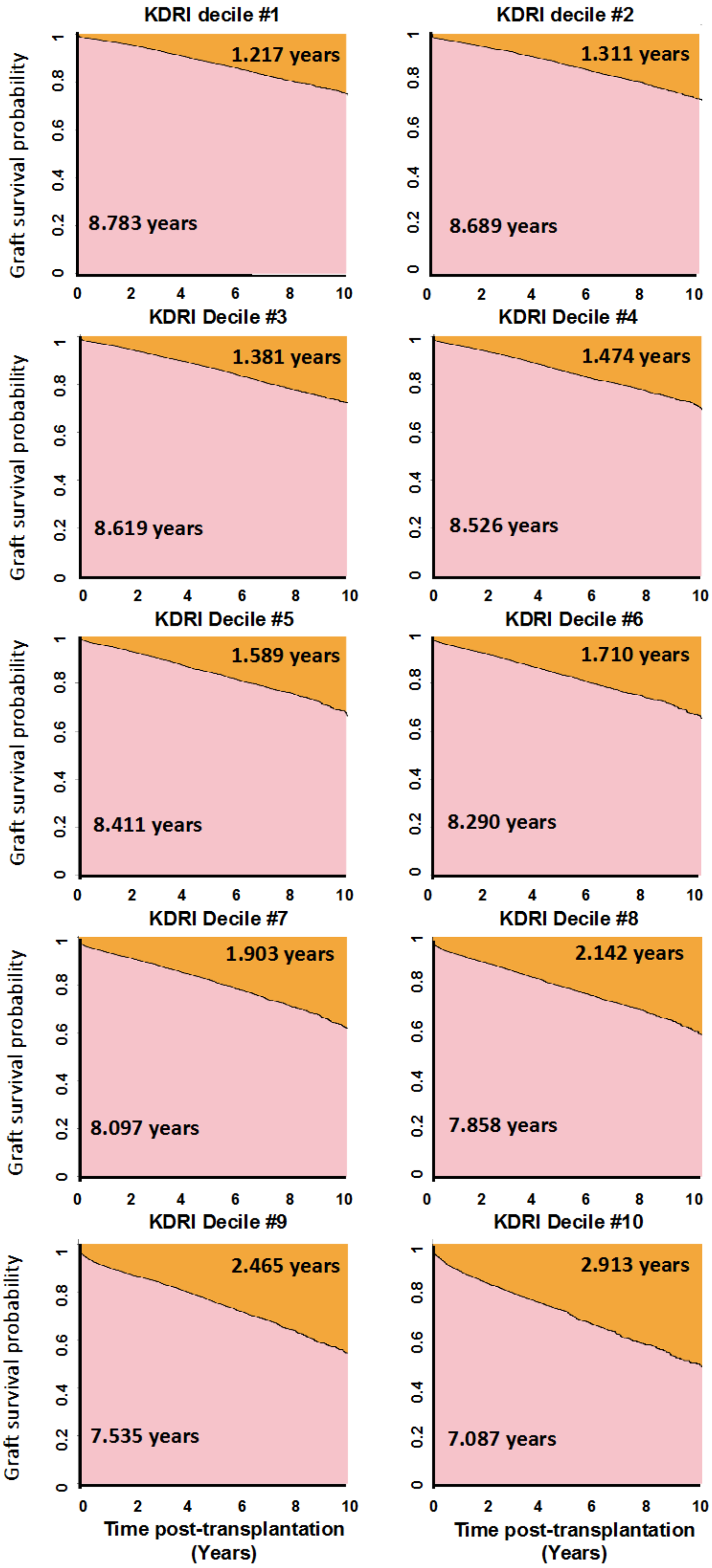

© 2019 Olivier A et al. *JAMA Internal Medicine*.

eFigure 8: Kaplan-Meier curves for kidney-graft survival, according to the deciles of the Kidney Donor Risk Index in France.

This figure shows the probability of graft survival, which is based on the KDRI deciles. The black curve corresponds to KDRI decile 1, the gray curve to KDRI decile 2, the green curve to KDRI decile 3, the light blue curve to KDRI decile 4, the dark blue curve to KDRI decile 5, the yellow curve to KDRI decile 6, the orange curve to KDRI decile 7, the pink curve to KDRI decile 8, the purple curve to KDRI decile 9, and the red curve to KDRI decile 10. The overall difference was significantly different (log-rank p<0.0001).

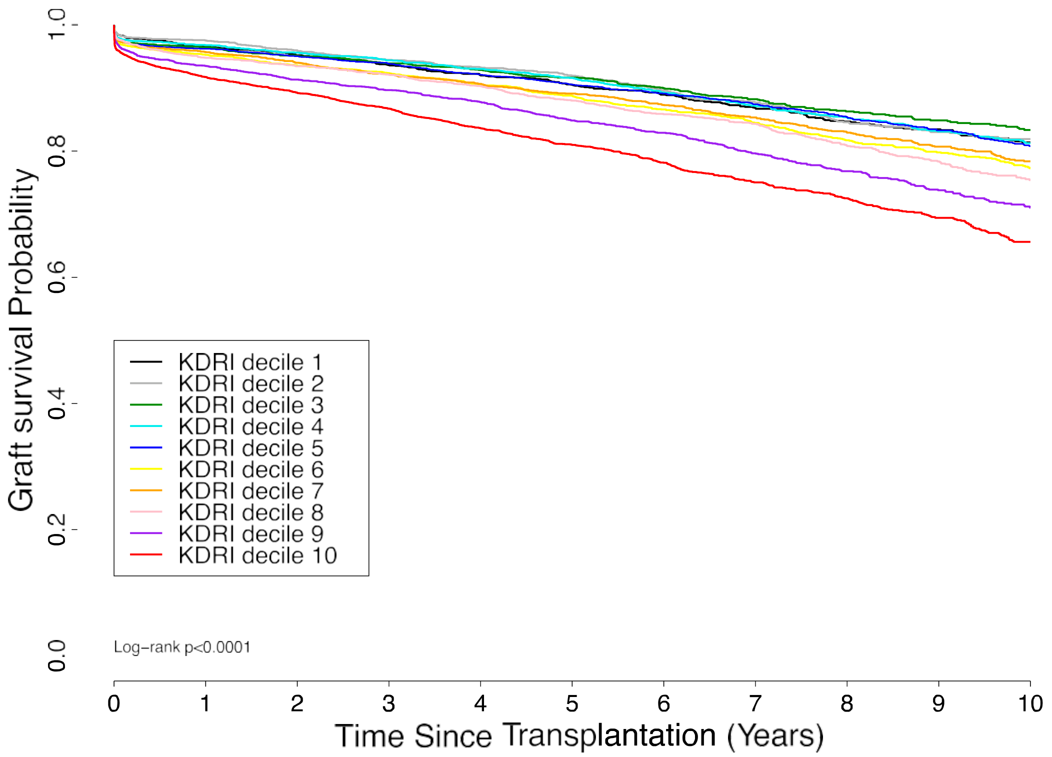

N at Risk											
KDR decile 1	1703	1618	1561	1515	1380	1243	1059	889	736	608	487
KDRI decile 2	1796	1711	1653	1594	1421	1258	1083	903	723	572	456
KDRI decile 3	1930	1819	1759	1695	1522	1356	1162	973	791	628	491
KDRI decile 4	2354	2224	2154	2084	1895	1698	1451	1187	964	758	601
KDRI decile 5	2880	2698	2615	2500	2245	1948	1658	1384	1133	910	694
KDRI decile 6	2940	2687	2568	2466	2200	1918	1624	1336	1086	847	625
KDRI decile 7	2802	2582	2468	2366	2097	1823	1567	1282	1040	809	593
KDRI decile 8	2809	2576	2446	2328	2049	1756	1474	1209	933	718	502
KDRI decile 9	3130	2748	2587	2431	2054	1658	1358	1063	834	620	428
KDRI decile 10	4908	4180	3848	3557	2866	2233	1698	1257	917	624	413

Abbreviations: KDRI; Kidney Donor Risk Index.

© 2019 Olivier A et al. *JAMA Internal Medicine*.

eFigure 9: Kaplan-Meier curves for non-death-censored kidney graft survival, according to the deciles of the Kidney Donor Risk Index in the US after transplantation.

This figure shows the probability of graft survival among actual kidney transplant recipients in the US and stratified by the KDRI deciles. The black curve corresponds to KDRI decile 1, the gray curve to KDRI decile 2, the green curve to KDRI decile 3, the light blue curve to KDRI decile 4, the dark blue curve to KDRI decile 5, the yellow curve to KDRI decile 6, the orange curve to KDRI decile 7, the pink curve to KDRI decile 8, the purple curve to KDRI decile 9, and the red curve to KDRI decile 10. The overall difference was significantly different (log-rank p<0.0001).

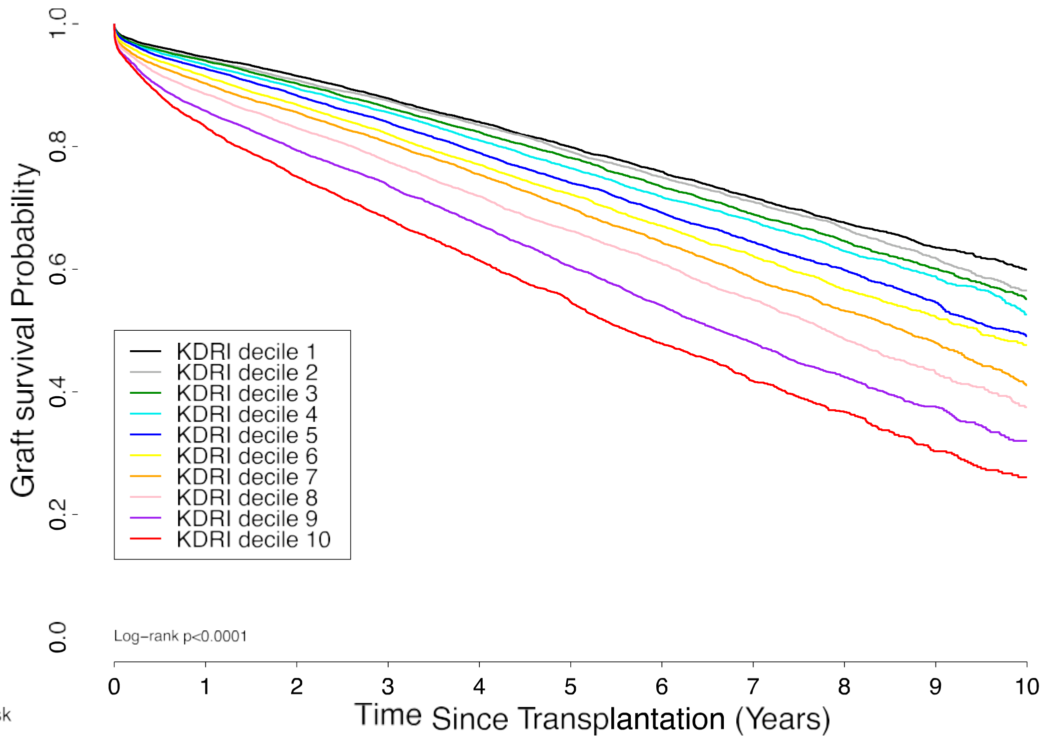

N at Risk	0	1	2	3	4	5	6	7	8	9	10
KDRI decile 1	15352	12703	10760	8854	7178	5651	4342	3192	2120	1182	396
KDRI decile 2	15089	12333	10382	8524	6724	5226	3893	2850	1820	985	327
KDRI decile 3	14859	12068	9977	8088	6414	4962	3695	2600	1659	880	307
KDRI decile 4	14634	11717	9640	7836	6114	4682	3412	2408	1504	819	256
KDRI decile 5	14082	11274	9211	7433	5872	4483	3267	2248	1460	782	274
KDRI decile 6	13194	10466	8606	6914	5425	4094	2932	2003	1211	610	218
KDRI decile 7	12215	9534	7752	6195	4822	3671	2641	1819	1120	601	218
KDRI decile 8	10863	8340	6719	5351	4149	3058	2187	1464	867	412	126
KDRI decile 9	9063	6865	5493	4391	3329	2425	1681	1046	634	311	97
KDRI decile 10	5445	4040	3244	2561	1956	1414	950	606	351	160	48

© 2019 Olivier A et al. *JAMA Internal Medicine*.

eFigure 10: Kaplan-Meier curves for non-death-censored kidney-graft survival, according to the deciles of the Kidney Donor Risk Index in France after transplantation. This figure shows the probability of graft survival among actual kidney transplant recipients in the France and stratified by the KDRI deciles. The black curve corresponds to KDRI decile 1, the gray curve to KDRI decile 2, the green curve to KDRI decile 3, the light blue curve to KDRI decile 4, the dark blue curve to KDRI decile 5, the yellow curve to KDRI decile 6, the orange curve to KDRI decile 7, the pink curve to KDRI decile 8, the purple curve to KDRI decile 9, and the red curve to KDRI decile 10. The overall difference was significantly different (log-rank p<0.0001).

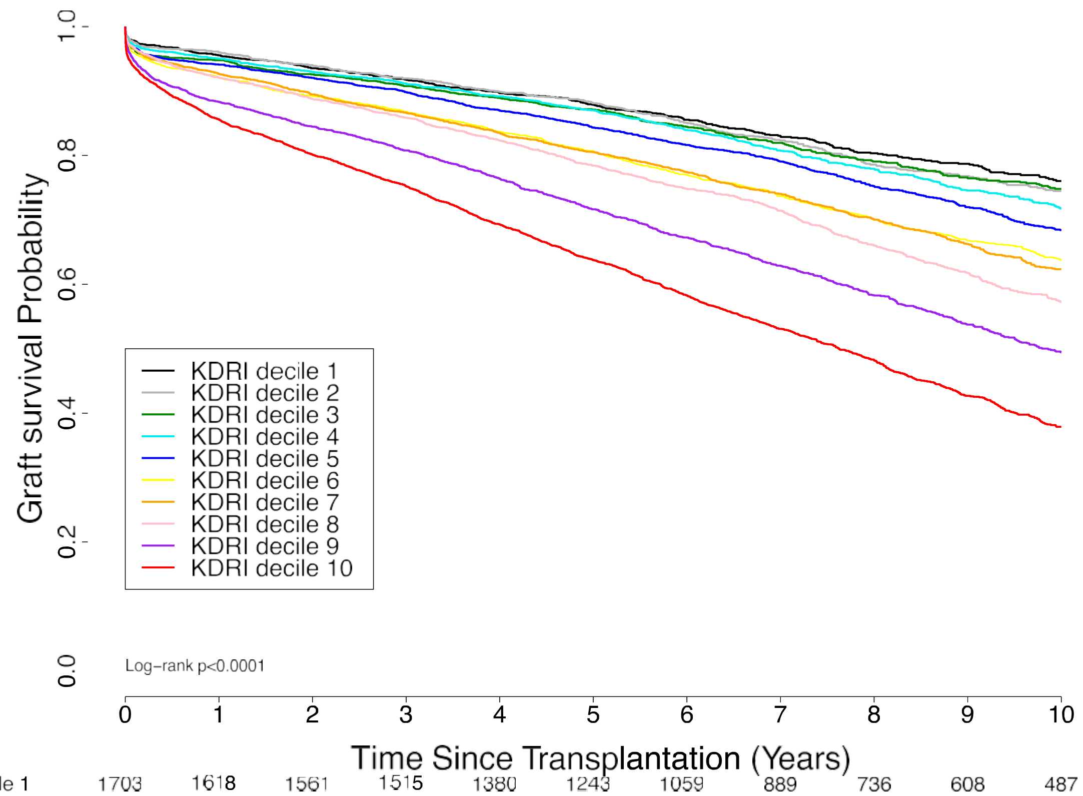

N at Risk											
KDRI decile 1	1703	1618	1561	1515	1380	1243	1059	889	736	608	487
KDRI decile 2	1796	1711	1653	1594	1421	1258	1083	903	723	572	456
KDRI decile 3	1930	1819	1759	1695	1522	1356	1162	973	791	628	491
KDRI decile 4	2354	2224	2154	2084	1895	1698	1451	1187	964	758	601
KDRI decile 5	2880	2698	2615	2500	2245	1948	1658	1384	1133	910	694
KDRI decile 6	2940	2687	2568	2466	2200	1918	1624	1336	1086	847	625
KDRI decile 7	2802	2582	2468	2366	2097	1823	1567	1282	1040	809	593
KDRI decile 8	2809	2576	2446	2328	2049	1756	1474	1209	933	718	502
KDRI decile 9	3130	2748	2587	2431	2054	1658	1358	1063	834	620	428
KDRI decile 10	4908	4180	3848	3557	2866	2233	1698	1257	917	624	413

© 2019 Olivier A et al. *JAMA Internal Medicine*.

eFigure 11: Estimation of gains in non-death-censored allograft life years from reducing high kidney discard rates in the US, according to KDRI categories.

Panel A shows the life years saved by decile of KDRI in the US if the French model of kidney acceptance had been used. The black curve corresponds to KDRI decile 1, the gray curve to KDRI decile 2, the green curve to KDRI decile 3, the light blue curve to KDRI decile 4, the dark blue curve to KDRI decile 5, the yellow curve to KDRI decile 6, the orange curve to KDRI decile 7, the pink curve to KDRI decile 8, the purple curve to KDRI decile 9, and the red curve to KDRI decile 10. The x-axis corresponds to the time post transplantation, and the vertical axis corresponds to the number of allograft life years saved. Panel B shows the life years saved overall if organ utilization patterns in the US had followed the French model. The x-axis corresponds to the time post transplantation, and the vertical axis corresponds to the number of allograft life years saved.

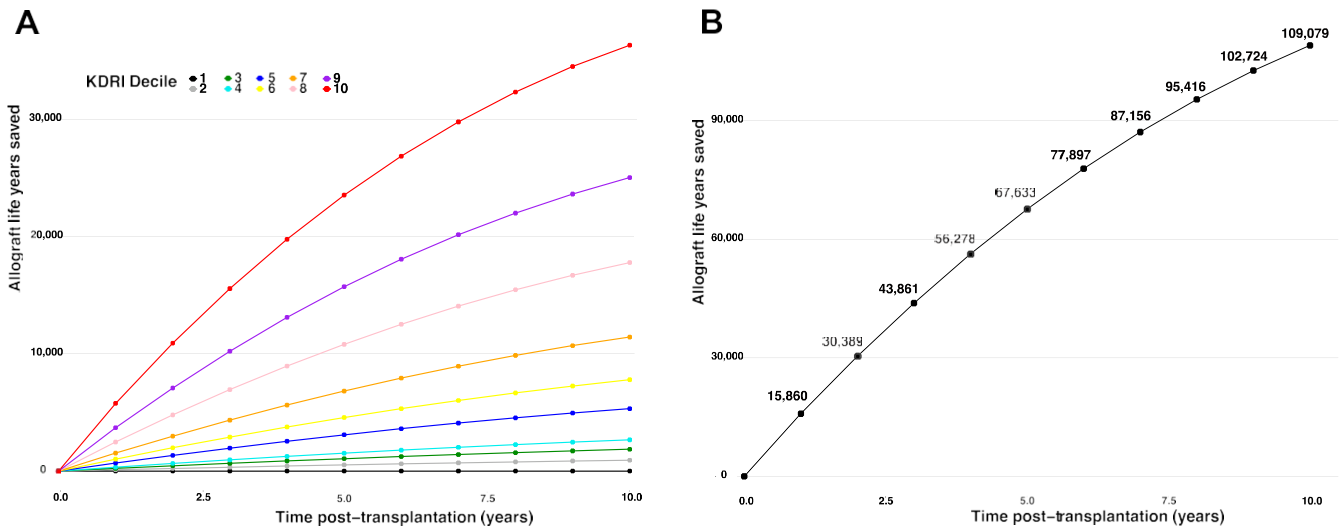

© 2019 Olivier A et al. *JAMA Internal Medicine*.

References

1. Rao PS, Schaubel DE, Guidinger MK, Andreoni KA, Wolfe RA, Merion RM, Port FK, Sung RS. A comprehensive risk quantification score for deceased donor kidneys: the kidney donor risk index. Transplantation. 2009;88(2):231-6. Epub 2009/07/23. doi: 10.1097/TP.0b013e3181ac620b. PubMed PMID: 19623019.
2. Organ Procurement and Transplantation Network. Kidney Donor Profile Index (KDPI) Guide for Clinicians. URL: https://optn.transplant.hrsa.gov/resources/guidance/kidney-donor-profile-index-kdpi-guide-for-clinicians/. Accessed 01/30/2017.

© 2019 Olivier A et al. *JAMA Internal Medicine*.
